# Stearoyl-CoA desaturase inhibition normalizes brain lipid saturation, **α**-synuclein homeostasis, and motor function in mutant Gba1-Parkinson mice

**DOI:** 10.1172/jci.insight.188413

**Published:** 2025-06-03

**Authors:** Silke Nuber, Harrison Hsiang, Esra’a Keewan, Tim E. Moors, Sydney J. Reitz, Anupama Tiwari, Gary P.H. Ho, Elena Su, Wolf Hahn, Marie-Alexandre Adom, Riddhima Pathak, Matthew Blizzard, Sangjune Kim, Han Seok Ko, Xiaoqun Zhang, Per Svenningsson, Dennis J. Selkoe, Saranna Fanning

**Affiliations:** 1 Ann Romney Center for Neurologic Diseases, Brigham and Women’s Hospital and Harvard Medical School, Boston, Massachusetts, USA.; 2 Department of Neurology and; 3 Neuroregeneration and Stem Cell Programs, Institute for Cell Engineering, Johns Hopkins University School of Medicine, Baltimore, Maryland, USA.; 4 Neuro Svenningsson, Department of Clinical Neuroscience, Karolinska Institutet, Stockholm, Sweden.

**Keywords:** Cell biology, Neuroscience, Parkinson disease

## Abstract

Loss-of-function mutations in the *GBA1* gene are a prevalent risk factor for Parkinson’s disease (PD). Defining features are Lewy bodies that can be rich in α-synuclein (αS), vesicle membranes, and other lipid membranes, coupled with striatal dopamine loss and progressive motor dysfunction. Of these, lipid abnormalities are the least understood. An altered lipid metabolism in PD patient-derived neurons — carrying mutations in either GBA1, encoding for glucocerebrosidase (GCase), or αS — shifted the physiological αS tetramer/monomer (T:M) equilibrium, resulting in PD phenotypes. We previously reported inhibition of stearoyl-CoA desaturase (SCD), the rate-limiting enzyme for fatty acid desaturation, stabilized αS tetramers and improved motor deficits in αS mice. Here we show that mutant GBA1-PD cultured neurons have increased SCD products (monounsaturated fatty acids [MUFAS]) and reduced αS T:M ratios that were improved by inhibiting SCD. Oral treatment of symptomatic L444P and E326K Gba1 mutant mice with 5b also improved the αS T:M homeostasis and dopaminergic striatal integrity. Moreover, SCD inhibition normalized GCase maturation and dampened lysosomal and lipid-rich clustering, key features of neuropathology in GBA-PD. In conclusion, this study supports that brain MUFA metabolism links *GBA1* genotype and WT αS homeostasis to downstream neuronal and behavioral impairments, identifying SCD as a therapeutic target for GBA-PD.

## Abstract 2

Our study shows that mutations in glucocerebrosidase 1 (GBA1) leading to an accumulation of monounsaturated fatty acid and other lipids in the brain are sufficient to cause destablilization of a-synuclein tetramers and downstream phenotypes that can be restored by inhibition a key fatty acid desaturase SCD.

## Graphical abstract

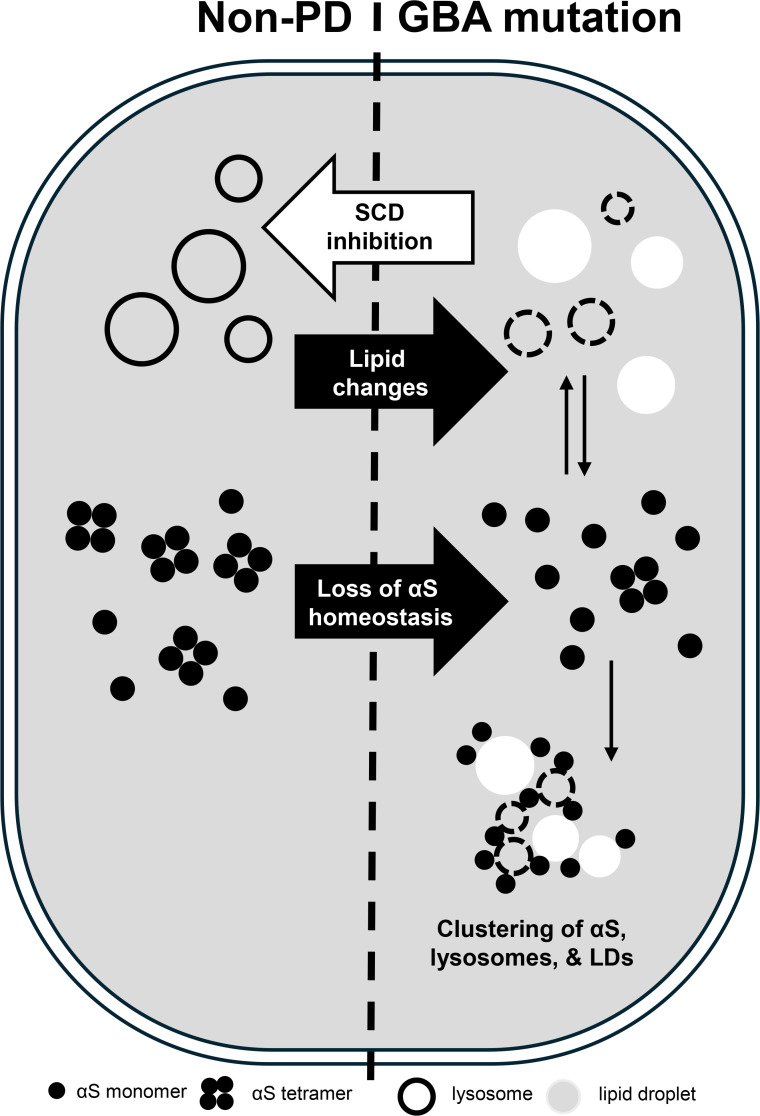

## Introduction

*GBA1* gene mutations are recognized as the most common genetic risk factor for developing Parkinson’s disease (PD) (1, 2). The gene encodes for the lysosomal lipid enzyme glucocerebrosidase (GCase). Homozygous GBA1 mutations can cause neuropathic Gaucher’s disease (GD). Both hetero- and homozygous GBA1 mutations reduce GCase enzyme level and activity, resulting in lysosomal dysfunction and pathologic accumulation in α-synuclein (αS) (3, 4). The degree of pathogenicity associated with each individual GBA1 mutation differs, and variants have been stratified into mild (e.g., N370S) or severe variants (e.g., L444P) (5–7). Interestingly, the GBA1 E326K variant is not associated with GD (8) but still constitutes a risk factor for PD, leading to motor and cognitive complications. The L444P mutation has a relative higher penetrance and a more severe PD development and is associated with neuronopathic GD (9, 10).

Experimental evidence from studies in mice and cells indicates that αS accumulation occurs secondary to pathogenic GBA1 mutations (3, 11–13). Pathologic αS aggregation can disrupt GCase trafficking and, thereby, its lysosomal function, forming a bidirectional loop (3). Accumulating evidence from our laboratory (14–18) and others (19–21) showed that αS can occur in an physiological equilibrium between helically folded tetramers (T) and natively unfolded monomers (M). Loss of this homeostasis is associated with disease; a decrease in the physiological T:M ratio has been described in all tested familial PD single-point mutations (15), SNCA triplication (22), and LRRK2 mutations (18). Accordingly, neurons harboring PD-causing GBA1 mutations shifted the endogenous (WT) αS T:M ratio accompanied by abnormal serine 129 phosphorylation and total αS accumulation (23), indicating that lipid metabolism can affect the physiological αS homeostasis. Overexpressing WT GBA1 improved the αS T:M ratio and PD-like motor phenotypes in tetramer-abrogating 3K (E35K, E46K, E61K) mutant αS mice (24). Likewise, treatment of GBA1 L444P mutant primary neurons and patient-derived neurons with miglustat augments GCase activity and reverses the destabilization of the αS tetramers (23). These observations strongly indicate that lipid metabolism can affect the αS homeostasis.

PD lipid pathology associating with αS aggregates were first observed by Lysia Forno, describing lipoid granules (lipofuscin) and a neuropathological hallmark of Lewy bodies (LBs) (25) and, more recently lipids, lipid-rich membranes and lysosomes were identified as a common component of LBs (26). Since 2000 accumulating reports described a relationship of unsaturated fatty acids and LBs, including biology of αS (27–29), epidemiology (30), and genetics (31, 32). αS has a similar motif homologous to that in fatty acid-binding proteins, allowing it to bind to oleic acid (33), which facilitates the formation of toxic oligomers (34). Recent lipidomic profiling data indicate a correlation between brain lipid accumulation and PD pathogenesis (35, 36), suggesting monounsaturated fatty acid metabolism as a therapeutical target for PD pathogenesis (e.g., recently reviewed by Fanning et al.; ref. 37).

One such therapeutical target is stearoyl-CoA desaturase (SCD), a key regulator of monounsaturated fatty acid (MUFA) synthesis. SCD has been reported to be upregulated together with MUFAs in Alzheimer’s disease (AD) (38–40). Accordingly, an increase in MUFAs have also been described in brains of AD with LBs (ADLB), PD, and other synucleinopathies (41), and SCD inhibition improved memory function in AD mouse models (42, 43). We have previously demonstrated that the in vitro knockdown of *SCD1* in human WT αS (34) or an engineered amplification of the familial PD E46K-type (3K) (44) neurons resulted in fewer αS aggregates and reduced cell death. Both knockdown SCD1 or treatment with brain-penetrant SCD inhibitors in 3K αS mutant mice showed a decrease in αS^+^ lipid rich aggregates and fiber degeneration, and these were coupled with more soluble αS tetramers and improved motor phenotypes (45, 46). Decreasing MUFAs via SCD inhibition also improved the αS T:M ratio in PD, causing SNCA triplication patient neurons (22) and LRRK2 mutant patient neurons (18). Notably, a SCD inhibitor has advanced into phase I clinical trial, and increasingly, more therapy research focused on the brain lipids is recognized (47–49).

Based on the emerging links between *GBA1* genotype and αS biology and on the previous success in reestablishing αS tetramers and normalizing phenotypes when inhibiting SCD, we applied a SCD inhibitor to GBA-PD patient-derived neurons and knock-in (KI) mice with decreased GCase activity and αS aggregation pathologies (50–54). Here we show that PD patient-derived GBA1 mutant neurons and homozygous Gba1-KI mice have increased level of MUFAs (measured by the C16 and C18 fatty acid desaturation index [FADI]), reduced αS tetramerization, and these are associated with motor deficits. Treatment with the brain-penetrant SCD inhibitor “5b” lowered the MUFA levels and increased the αS T:M ratios. In Gba1 mice, the 5b treatment reduced the lipid-rich and proteinase K-resistant (PK-resistant) phosphorylated αS–positive (pS129^+^) aggregates and improved lysosomal biogenesis and distribution and dopaminergic (DAergic) integrity. Beside MUFAs, lipidomic profiling of Gba1 mutant mouse brain revealed an abnormally increased level of unsaturated and oxidized membrane phospholipids, in particular PEs, that were also reduced by SCD inhibition.

Together, these data support a hypothesis of MUFA-dependent regulation of brain αS homeostasis, including T:M equilibrium in PD and GBA1-associated PD.

## Results

### GBA1 L444P PD patient-derived cortical neurons have increased total C16:1 and C18:1 fatty acid associating with decreased αS T:M ratio that are rescued by pharmacological inhibition of SCD.

**Figure 1:**
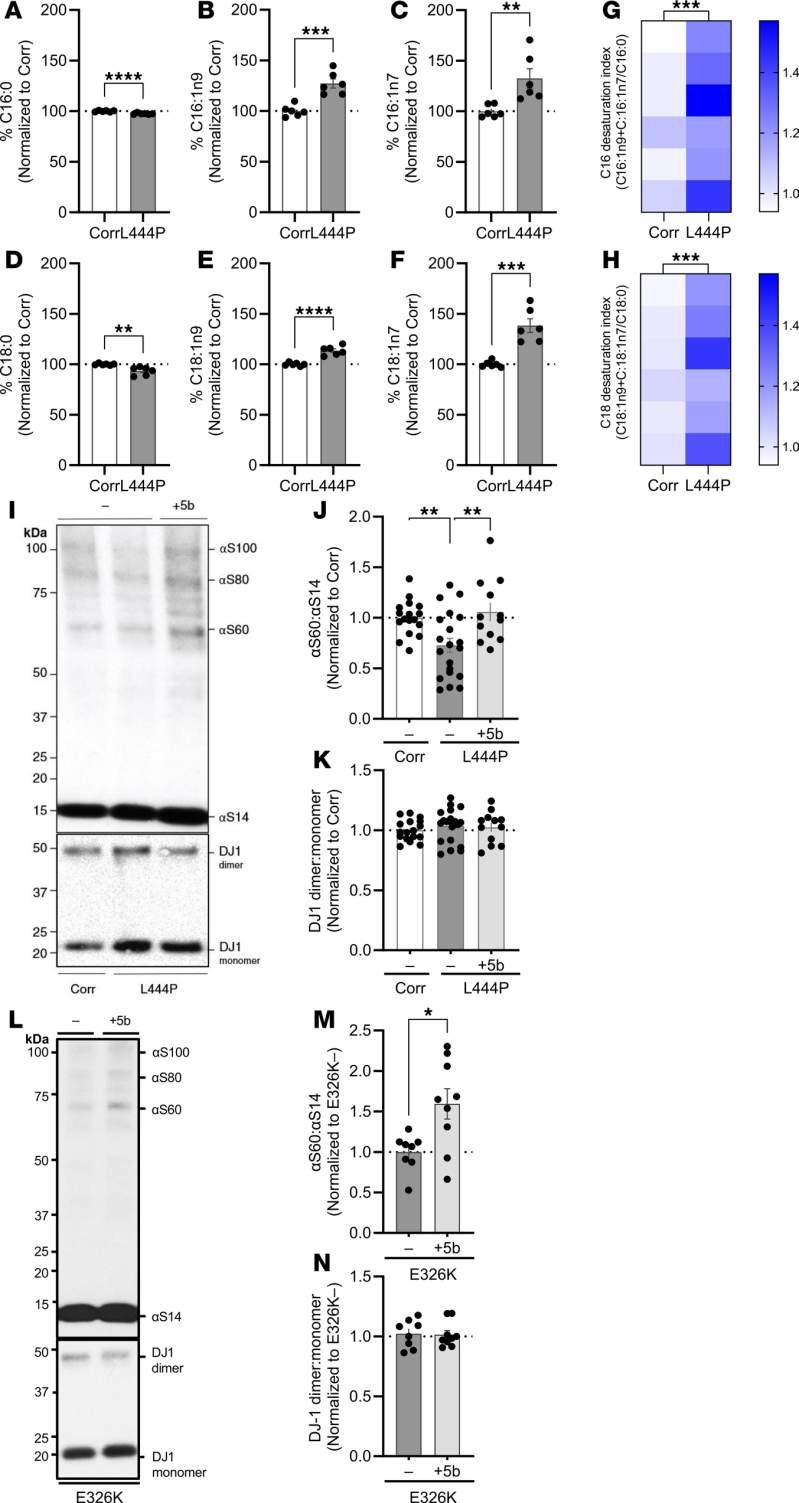
Monounsaturated fatty acids (C16:1 and C18:1) are increased in GBA1 L444P mutant iPSC neurons and SCD inhibition increases αS T:M ratio of patient-derived GBA1 L444P and E326K mutant iPSC neurons. GBA L444P mutant and isogenic corrected neurons were differentiated (DIV 20) and harvested for FA analysis by gas chromatography. *n* = 6. (**A**–**C**) Total cellular C16:0, C16:1n9, and C16:1n7 of GBA1 L444P mutant neurons and isogenic corrected control neurons were measured by gas chromatography. Data are reported relative to the isogenic corrected line. GraphPad Prism 10, unpaired 2-tailed *t* test. (**D**–**F**) Total cellular C18:0, C18:1n9, C18:1n7 of GBA1 L444P mutant neurons and isogenic corrected control neurons were measured by gas chromatography. Data is reported relative to the isogenic corrected line. Statistical analysis: Graphpad Prism 10, unpaired 2-tailed *t* test. (**G**) Heatmap shows the calculated desaturation index for C16:1n9+C16:1n7/C16:0 for GBA L444P mutant neurons versus isogenic corrected control neurons. (**H**) Heatmap shows the calculated desaturation index for C18:1n9+C18:1n7/C18:0 for GBA L444P mutant neurons versus isogenic corrected control neurons. (**G** and **H**) *n* = 6. (**I** and **L**) L444P and E326K neurons were treated with 1 μM 5b or DMSO. Cells were crosslinked using DSG. Cell lysates were immunoblotted to detect and quantify αS14, αS60, and DJ-1 (crosslinking control). (**J**) L444P: Quantification of αS60:αS14 (T:M) ratio. Two-way ANOVA: statistically significant effects of condition (*F*_1,46_ = 18.75, *P* = 0.0011) and treatment (*F*_1,46_ = 21.69, *P* = 0.0005), Tukey’s’s HSD test, Corr without 5b (Corr–5b) versus L444P–5b, *P* = 0.0060, L444P–5b versus L444P with 5b (L444P+5b), *P* = 0.0028; *N* = 2, *n* = 12–19). (**M**) E326K: Quantification of αS60:αS14 (T:M) ratio. (**K** and **N**) No statistical differences in DJ-1 (*P* > 0.05). Data are shown as mean ± SD. Unpaired 2-tailed *t* test, *P* = 0.0036; *N* = 1, *n* = 8–9. *P < 0.05, **P < 0.01, ***P < 0.001, ****P < 0.0001.

We and others previously identified a primary α-synucleinopathy associated with increased cellular MUFAs (C16:1, C18:1) (22, 55, 56). Correcting this MUFA imbalance with SCD inhibitor treatment reversed PD-relevant phenotypes in vitro and in vivo, and an SCD inhibitor subsequently entered PD clinical trials (22, 44–46, 55, 57, 58). Here, we hypothesized that there may be increased MUFAs in a nonprimary α-synucleinopathy, resulting from mutations associated with GBA1-PD. To assess MUFAs in GBA1 mutation carriers, we performed a fatty acid profiling comparing patient-derived GBA1-PD (L444P) mutant iPSC neurons with those of an isogenic corrected control. Analysis focused on MUFAs C16:1 (n9, n7) and C18:1 (n9, n7), and their saturated chain length matched precursors C16:0 and C18:0. Both forms of C16:1 (n9, n7) were increased in the L444P neurons relative to the isogenic control neurons with a small decrease (as expected) for the saturated precursor C16:0 (Figure 1, A–C) (C16:0, *P* < 0.0001; C16:1n9, *P* = 0.0003; C16:1n7, *P* = 0.0081). Similarly, both C18:1n9 and C18:1n7 were increased in the mutant neurons relative to the isogenic control neurons with decreased C18:0 (Figure 1, D–F) (C18:0, *P* = 0.0028; C18:1n9, *P* < 0.0001; C18:1n7, *P* = 0.0002). This increase in MUFAs and decrease in saturated fatty acids C16:0 and C18:0 resulted in an overall increase in the desaturation index (Figure 1, G and H) (C16:1/C16:0, *P* = 0.0007; C18:1/C18:0, *P* = 0.0004). Based on these substantial increases in SCD-related C16 and C18 FADI, we hypothesized correcting MUFA dyshomeostasis via SCD inhibition as a candidate therapeutic approach for GBA1 mutation carriers.

We previously showed that a brain-penetrant SCD inhibitor (5b) corrected the aberrant αS T:M ratio in 3K mice (46) and PD patient-derived αS triplication neurons (22). Here, we investigated 5b treatment of GBA1 L444P and E326K patient-derived cortical neurons. The cells were treated with 1 μM 5b and crosslinked using DSG. In line with the findings of Kim and colleagues (23), the αS T:M ratio was markedly reduced in L444P neurons relative to that of the isogenic corrected neurons (2-way ANOVA, *F*_1,46_ = 18.75, *P* = 0.0011). The L444P-associated αS T:M was normalized by 5b treatment (*P* = 0.0028; Figure 1, I and J). 5b treatment significantly increased αS T:M ratio to a similar degree in GBA1 E326K neurons (unpaired 2-tailed *t* test, *P* < 0.01; Figure 1, L and M). No differences were detected in DJ-1, which served as loading and crosslinking quality control (Figure 1, K and N). Quantifying αS tetramers plus probable higher molecular weight conformers of the tetramer (potential octamers and hexamers at ~80 and 100 kDa) (59) revealed a significant decrease in L444P versus isogenic corrected controls (*P* < 0.05), and 5b treatment raised the multimer/monomer ratio (*P* < 0.05) (Supplemental Figure 1, A and B; supplemental material available online with this article; https://doi.org/10.1172/jci.insight.188413DS1). Previous studies show that GBA1 L444P–mediated GCase deficiency (50) negatively affects the αS T:M ratio in brain neurons (23). We next tested GCase activity in GBA1 E326K neurons and observed a significant reduction in activity at pH 4.5 (*P* < 0.0001; Supplemental Figure 1C).

These data suggest that GCase-deficient GBA-PD–derived neurons with increased SCD products have a decrease in the physiological αS tetramerization that can be increased by SCD inhibition.

### Gba1 mutations cause a progressive motor syndrome associated with a decrease in mature GCase and the αS T:M ratio in mice.

Previous studies have reported that homozygous E326K Gba1 KI mice develop progressive neuropathology, including a decrease in GCase activity, tyrosine hydroxylase^+^ (TH^+^) neuronal loss, and a higher susceptibility toward fibrillar αS, associating with an age-dependent motor deficit (60), while heterozygous E326K or L444P mice show only very subtle changes (13, 60). In order to promote phenotype development, we bred both L444P and E326K mouse lines to homozygosity. Previously, these L444P (The Jackson Laboratory, 024574) and E326K KI mice were reported to be viable and fertile and to live a normal life span (13, 60). Accordingly, we did not observe any changes in viability or fertility between the homozygous Gba1 KI mouse lines and control (Ctl) mice (data not shown).

**Figure 2:**
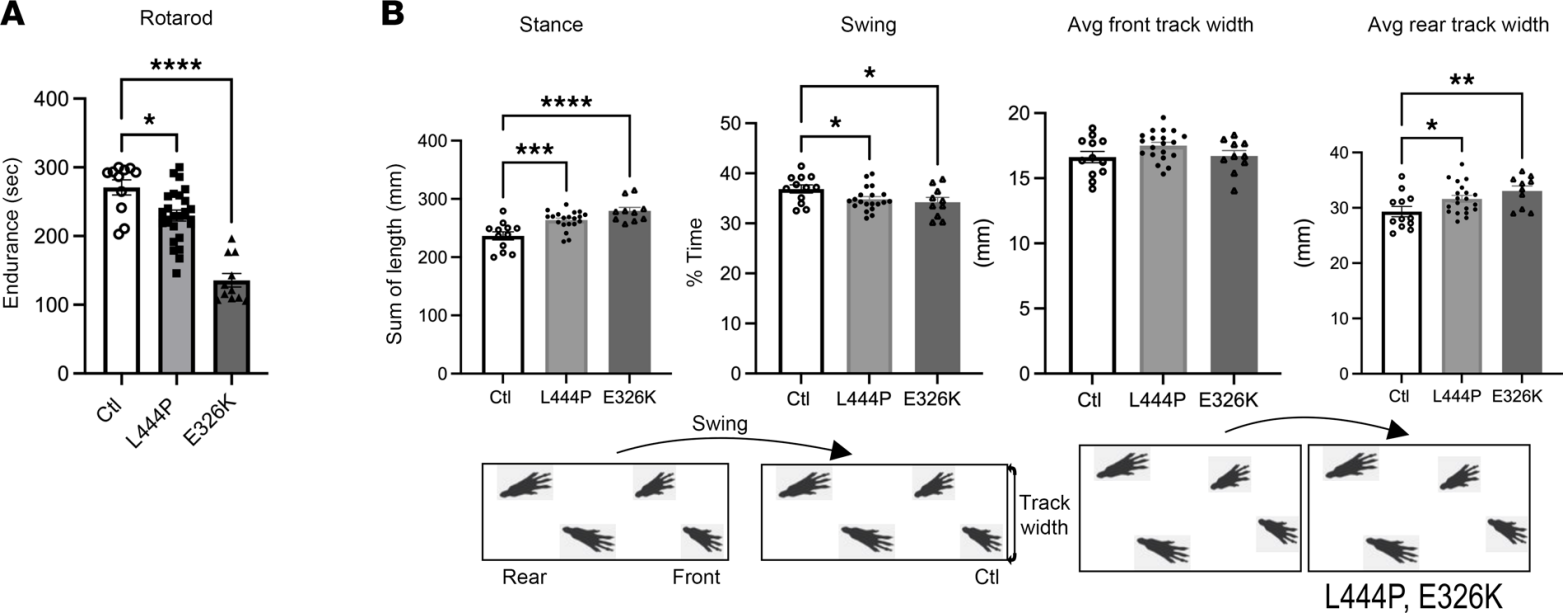
Gba1 mutant mice develop a progressive motor decline and an insecure gait. (**A**) Graph quantifies balancing on a 4–40 rpm accelerating rotarod (avg. of 6 trials on 3 consecutive days) at 12 months. (**B**) Automated gait scans of mouse paw pattern on a horizontal treadmill (Cleversys) at 12 months. Schematic of stepping pattern derived from gait scans display insecure (bradykinetic) gait in Gba1 mutant mice. Data are shown as mean ± SEM. *P < 0.05, **P < 0.01, ***P < 0.001; ****P < 0.0001. One-way ANOVA, Tukey’s post hoc test.

Accelerated rotarod testing (4–40 rpm, average of 6 testing trials during 3 consecutive days [d]) of 12-month-old L444P and E326K mice showed a significant decrease versus age-matched Ctl mice (Ctl 262 ± 10 seconds; L444P 234 ± 7 seconds; E326K 135 ± 10 seconds; 1-way ANOVA; *P* < 0.0001). To more finely analyze the underlying gait changes at 12 months, Gba1 mutant and Ctl mice were additionally subjected to a gait scan analysis, using high-resolution photography of gait symmetry by placing mice on a transparent motoric belt. A one-way ANOVA showed an increase in stance (*P* < 0.0001), a shorter percentage of time in limb swing (*P* < 0.04) and an increase in the average rear track width (*P* = 0.01), but no changes were observed front track width (Figure 2B). Together, these data suggest motor deficits and an abnormal, insecure gait (paw-to-body support) in aged (12 months) Gba1 mutant mice.

**Figure 3:**
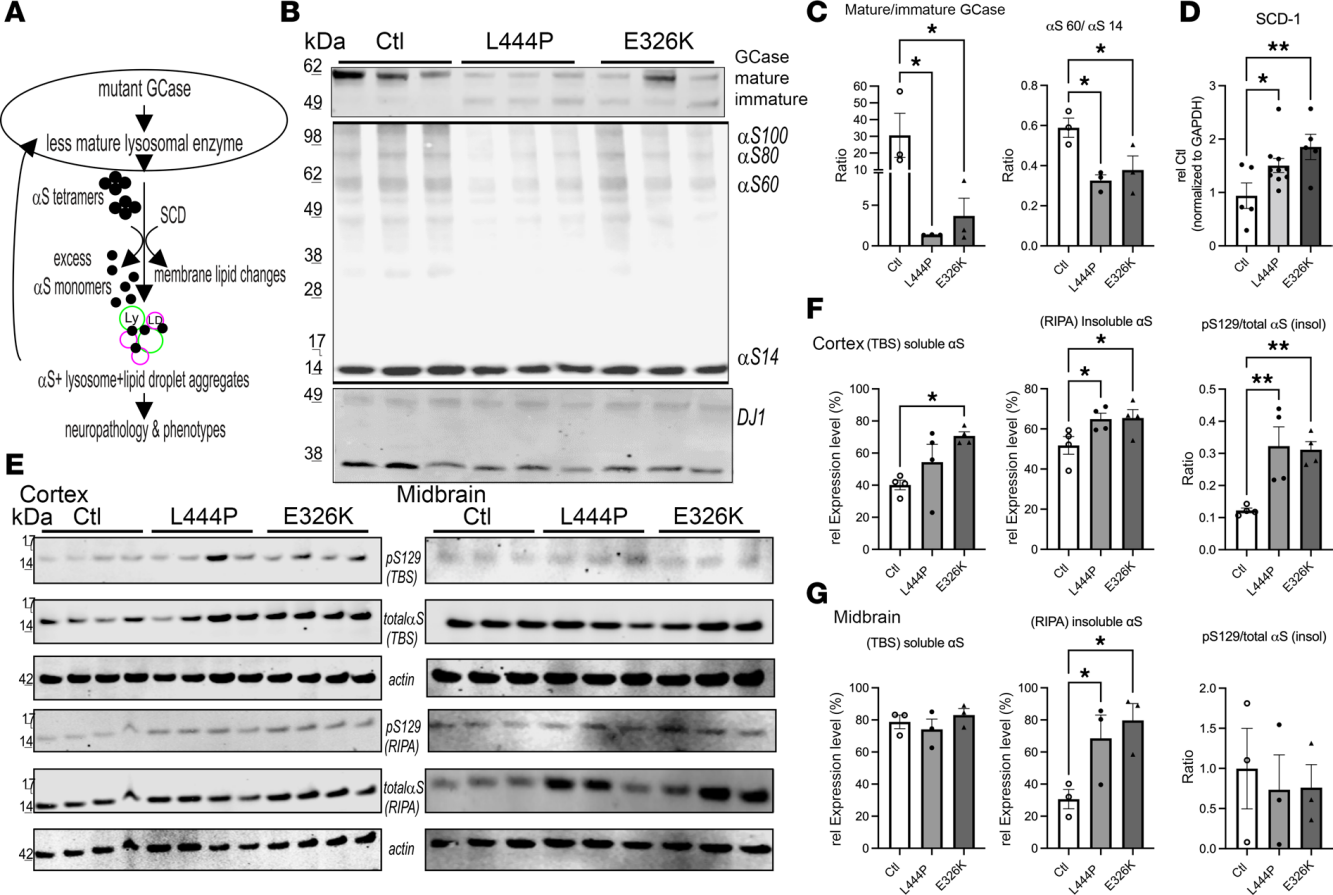
Gba1 mutant mice display a decrease in GCase maturation, a decrease in the αS T:M ratio, and more insoluble pS129^+^ mice. (**A**) Lysosomal dysfunction hypothesis. Reduced GCase associates with a disequilibrium of saturated and unsaturated FA in lysosomal and other phospholipid membranes leading to a shift in αS tetramers and the free monomers abnormally aggregate with lysosomes and lipid droplets, contributing to neuropathology and phenotypes. The αS^+^ aggregates can further decrease the amount of lysosomal active (mature) GCase. (**B**) WB of intact-cell crosslinked GCase and αS using cell-penetrant cross-linker DSG in cortical brain bits lysed with 1% Triton X-100/PBS. GCase signals reveal less mature (>62 kDa), more immature GCase (<62 kDa), and a reduced αS T:M signal in Gba1 mutant mice. DJ 1 signal serves as a control for crosslinking (dimer/monomer ratio) and loading. (**C**) Quantifies WBs in **B**. (**D**) Expression data from mouse SCD1 RNA in Ctl, L444P, and E326K (see also Supplemental Figure 2D). (**E**–**F**) WBs (noncrosslinked) of sequentially extracted TBS (soluble), RIPA-soluble (insoluble) extracts from cortex (left panels) and midbrain (right panels) (**E**), quantified in **F** and **G,** respectively. The TBS fraction was developed against (total) αS and the detergent-insoluble (RIPA) fraction against serine 129 phosphorylated αS and (total) αS of the corresponding blot. Actin serves as loading control. Data are shown as mean ± SEM. One-way ANOVA, Tukey’s post hoc. *P < 0.05, **P < 0.01.

GCase activity is reduced in L444P (52) and in E326K (60) mice. We hypothesized that reduced GCase function contributes to a disequilibrium of saturated and MUFAs (Figure 1). These MUFAs may then be incorporated into lysosomal and other phospholipid membranes, leading to abnormal αS membrane interactions that decrease αS tetramerization and allowing the resultant free monomers to aggregate with lysosomes and lipid droplets (LDs), inducing neuropathology and phenotypes. An accumulation of αS aggregates can further trigger a decrease in mature/functional GCase, creating a bidirectional loop (3) (Figure 3A).

The presence of lysosomally active GCase can also be inferred from the relative level of its biochemically higher molecular weight mature (glycosylated) form (>60 kDa, located at the lysosome) versus lower molecular weight immature forms (50–60 kDa, located at the endoplasmatic reticulum) (61). Quantitative Western blots (WBs) of the DSG-crosslinked cortical protein extracts showed a decrease of mature (>60 kDa) GCase and more immature (<60 kDa) GCase in both the L444P and the E326K mice (Figure 3, B and C). Reductions in GCase activity were also observed via a GCase activity assay, which is more sensitive than the relative GCase glycosylation (62), confirming reduced GCase activity in L444P and in E326K mouse cortex (Supplemental Figure 2A). Next, we evaluated the relative level of αS 60 tetramers to αS14 monomers (T:M) from the same protein extract, revealing a significant decrease in L444P and E326K mouse brain (Figure 3, B and C) (1-way ANOVA, *P* < 0.05) . Quantifying αS tetramers plus probable conformers (59) of the tetramer (potential octamers and hexamers) revealed a significant decrease in L444P (*P* < 0.05) and a trend toward a decrease in E326K (*P* = 0.08) (Supplemental Figure 2B). We next measured whether Gba1 mutations affect SCD expression, which may account for the detected increase in MUFAs in culture (Figure 1). The SCD1 mRNA level was increased in L444P and E326K mouse brain (Figure 3D and Supplemental Figure 2D).

Sequential extractions of brain cortices revealed an increase in (TBS) buffer-soluble and buffer-insoluble (RIPA) αS in Gba1 mutant mouse brain (Figure 3E). Buffer insolubility was confirmed in a second PD-vulnerable brain region, the midbrain (Figure 3E). We probed the insoluble lysates against phosphorylated serine (pS) 129 αS, an established marker for αS aggregation (63), and found that it was elevated in both the cortical and midbrain region of Gba1 mutant mice (Figure 3E). Given the accumulation in total insoluble αS, we quantified the pS129 αS/total αS ratio. Despite the overall accumulation of αS monomers, there was still a significant increase in pS129/total αS in brain cortex (1-way ANOVA, *P* < 0.03) (Figure 3F). There was no statistically evident change in the pS129/total αS ratio in the midbrain, which may be due to technical variability when dissecting this small brain region (Figure 3G).

### Brain FADI correction induced by 5b improves motor phenotypes and DAergic fiber integrity in Gba1 mutant mice.

**Figure 4:**
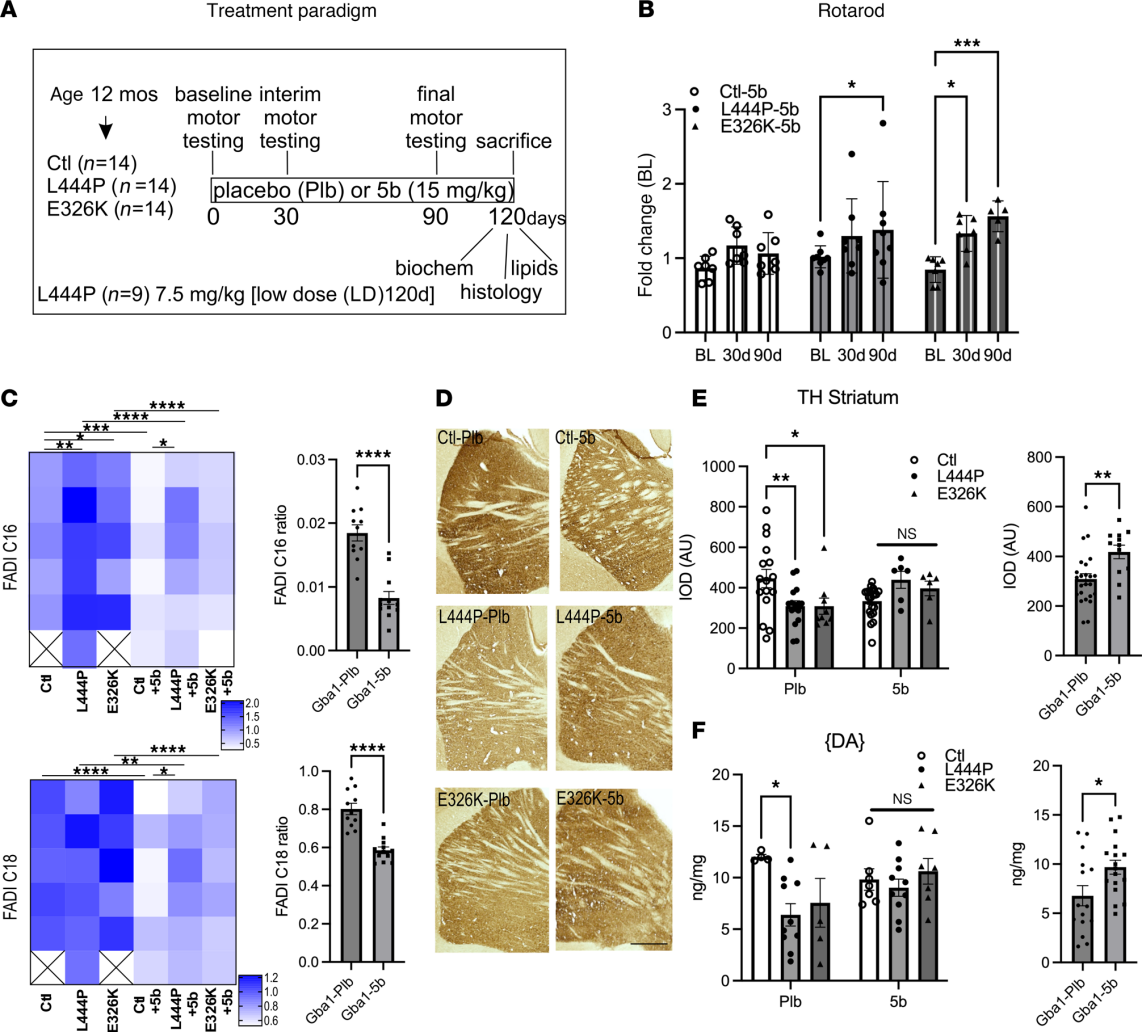
5b SCD inhibition improves the motor performance, lowers the fatty acid desaturation index, and restores striatal DAergic fiber densities and DA level. (**A**) SCD-inhibitor 5b treatment study of symptomatic (12 months old) L444P and E326K Gba1 versus Ctl mice. All mice were treated either with 15 mg/kg 5b or Plb. Some additional L444P Gba1 mice were treated with 7.5 mg/kg 5b (low dose [LD]; see Supplemental Figure 3). (**B**) Graph quantifies balancing skill learning on a 4–40 rpm accelerating rotarod. (**C**) Fatty acid saturation indices in brain cortex of 5b versus Plb treated mice validating efficacy reducing the specific MUFAs (C16:1, C18:1 versus C16:0, C18:0) ratio by 5b treatment (see also Supplemental Figure 1, D and E). Heatmaps show the calculated desaturation index for GBA L444P and E326K Plb and 5b. Note: Planned pairwise comparisons showed a relative increase of FADI C16:1/C16:0 in Plb L444P and E326K versus Ctl. Quantifying FADI C16 and FADI C18 in Gba1 (E326K+L444P) showed a significant decrease in 5b versus Plb. (**D**) Representative images of TH^+^ nerve terminals and fibers of Ctl, L444P, and E326K Gba1 mice. Scale bar: 600 μm. (**E**) Relative TH optical density (total of 12 sections; *n* = 3–4 mice each cohort) was analyzed in the dorsal striatum. (**F**) HPLC assay of striatal dopamine measured by HPLC. Data are shown as mean ± SEM. Two-way ANOVA with Bonferroni (**C**) or Tukey’s (**B**, **E**, and **F**) post hoc tests. Two-tailed, unpaired 2-tailed *t* test comparing Gba1 (E326K+L444P) Plb versus 5b. *P < 0.05, **P <0.01, ***P < 0.001, ****P < 0.0001

Based on the in vitro efficacy of 5b in GBA1-PD patient-derived iPSC neurons, we tested in vivo whether long-term 5b treatment could ameliorate the observed motor phenotypes, GCase maturation, and αS dyshomeostasis in Gba1 mutant mice. We used 12-month-old symptomatic Gba1 mutant mice (Figure 2A) and fed these mice with 5b formulated in chow (0.15 g per kg/food) ad libitum from age 12–16 months (Figure 4A). A subset of L444P mice were fed with a low dose (0.075 g per kg/food) of 5b (termed “L444P-LD-5b” (Supplemental Figure 3). Baseline (BL), interim (30d) and final (90d) motor assessment were performed, and the study ended after 120d (~16 weeks). An average chow intake of ~3 g/day was maintained throughout the study, indicating a daily dose intake of ~15 mg/kg. During the 4 months of treatment, 30d and 90d were selected to compare the motor performance in 5b-treated Gba1 mice versus placebo-treated (Plb-treated; diet without 5b) mice, and the 5b treatment led to a consistent improvement in GBA mutant mice (Figure 4B). Two-way ANOVA revealed a significant treatment effect over time (*F_2,55_* = 9.3*; P* < 0.001) and pairwise comparisons showed a statistical significance was achieved in both Gba1 mutant mouse lines at 90d (*P* < 0.05) (Figure 4B). While no improvement in the overall motor performance was detected in L444P-LD-5b (at 90d) (Supplemental Figure 3A), the mice displayed an improvement in balancing skills between trial 1 and trial 2 of the rotarod testing (Supplemental Figure 3B). This indicates enhanced motor skill learning in the low-dose–treated L444P versus Plb L444P similar to the improvements detected in the high-dose L444P-5b–treated mice (Supplemental Figure 3B).

Sixteen weeks after initiating 5b administration, mice were euthanized, and brains were harvested. The effect of SCD inhibition was confirmed by measuring the C16 and C18 FADI using liquid chromatography-mass spectrometry (LC-MS). Pairwise comparisons revealed a significant increase in the C16:1/C16:0 FADI in Plb Gba1 versus Ctl mouse brain, similar to changes detected in GBA1-PD patient-derived cortical neurons (Figure 1, A–C, and G) (Supplemental Figure 1, D and E). The 5b treatment normalized the C16 FADI in Gba1 mutant mice, consistently detected in all 5b-treated (Ctl, L444P and E326K) versus Plb-treated mouse cortices (2-way ANOVA, *P* < 0.0001) (Figure 4C). Testing for 5b treatment effects in Gba1 (L444P+E326K, combined) mice revealed a significant decrease in both the FADI C16 and FADI C18 by 5b (*P* < 0.0001; Figure 4C). Consistently, 5b treatment normalized the SCD1 RNA level between Ctl and Gba1 mutant mice and decreased the level comparing Gba1 (E326K+L444P, combined) Plb versus 5b (*P* < 0.0001) (Supplemental Figure 2D).

Given the improvement in motor phenotypes with 5b treatment in Gba1 mutant mice, we next determined the effects of 5b on PD-like brain DAergic pathologies, by assessing striatal TH immunoreactivity and striatal DA level. We quantified TH immunopositive nerve terminals in the dorsal striatum (caudate putamen), which is rich in projections from DAergic neurons in the substantia nigra pars compacta. Quantification in L444P and E326K showed a ~15% decrease in TH^+^ fibers versus Ctl at the analyzed age of 16 months (genotype × treatment, *P* = 0.001) and pairwise comparisons showed 5b-treated Gba1 (L444P and E326K) mice displayed no differences in TH^+^ neurites when comparing with 5b-treated Ctl mice (Figure 4, D and E). Testing for 5b treatment effects in Gba1 (L444P+E326K, combined) mice showed a significant increase in 5b versus Plb (*P* < 0.01) (Figure 4E). Accordingly, a lower striatal DA concentration was measured by HPLC in Gba1 mutant versus Ctl mice, reaching significance in L444P (*P* = 0.015), which was not detected when mice were treated with 5b (Figure 4F). The 5b treatment effects in Gba1 (L444P+E326K, combined) mice showed significantly increased DA level in Plb versus 5b (*P* < 0.05) (Figure 4F). The DA level was also similar between L444P-LD-5b, L444P-5b, and Plb control mice (Supplemental Figure 3C).

Having determined that 5b treatment effectively decreased brain SCD products and normalized striatal DAergic fiber integrity, we next investigated GCase maturation and αS homeostasis in the Plb- and 5b-treated Gba1 mice that had shown motor improvements over the Plb at 90d of treatment (Figure 4B).

**Figure 5:**
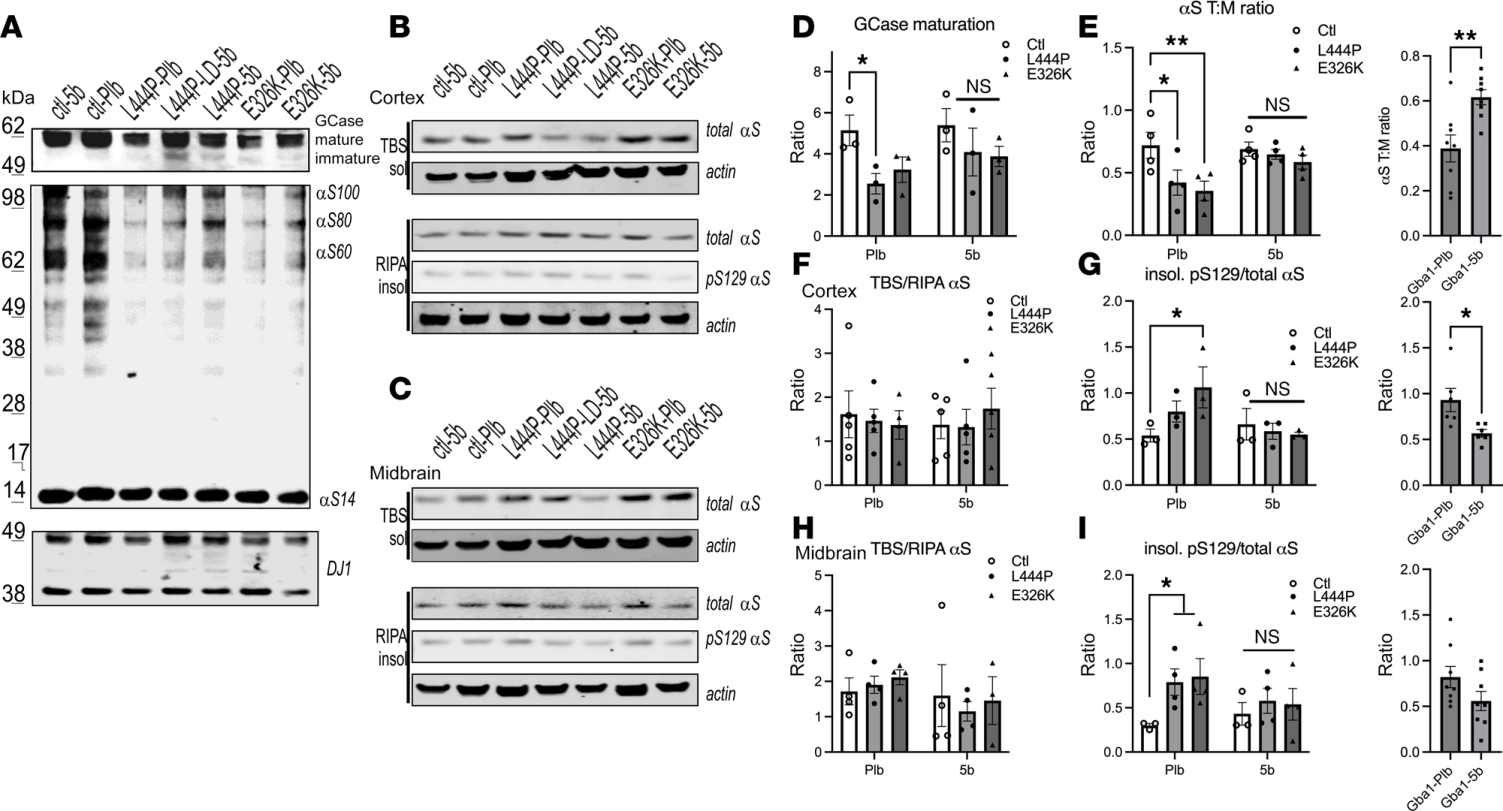
5b SCD inhibitor 5b normalizes GCase maturation, α**S T:M ratio and insoluble pS129 positivity in Gba1 mutant mice.** (**A**) Representative WB of GCase and αS using cell-penetrant cross-linker DSG in cortical brain bits lysed with 1% Triton X-100/PBS. 5b normalizes ratio of mature (>62 kDa) versus immature (<62 kDa) GCase (upper blot). 5b restores the αS T:M signal in GBA mutant mice (lower blot). DJ 1 signal serves as a control for crosslinking (dimer/monomer ratio) and loading. (**B** and **C**) Representative WBs (noncrosslinked) of sequentially extracted TBS (soluble), RIPA-soluble (insoluble) extracts from cortex and midbrain. (**D** and **E**) Quantified for mature/immature GCase and αS 60:14 kDa (T:M) ratio. (**F**–**I**) Quantified for TBS/RIPA-αS solubility and pS129/total αS in the insoluble (RIPA) extract in cortex and in midbrain. Data are shown as mean ± SEM. Two-way ANOVA, Tukey’s post hoc test (**D** and **E**, left panels). Two-tailed, unpaired 2-tailed *t* test comparing Gba1 (E326K+L444P) Plb versus 5b (**E**, **G**, and **I**, right panels). *P < 0.05, **P < 0.01.

First, we assessed the level of mature and immature GCase, which was lower in Plb Gba1 mutant mice, reaching significance in L444P versus Ctl (*P* = 0.03). However, these differences were not detected after 5b treatment in Gba1-5b versus Ctl-5b (Figure 5, A and D). Comparing Gba1 (L444P+E326K, combined) Plb versus 5b showed a nonsignificant trend in improved GCase maturation (*P* = 0.1) (data not shown). The αS T:M ratio revealed a significant decrease in Plb Gba1-mutant versus Plb-Ctl (2-way ANOVA; genotype, *P* = 0.02; treatment, *P* = 0.03), but no changes were detected between Ctl and Gba1 mutant mice after 5b treatment. The αS T:M level significantly raised when comparing Gba1 (L444P+E326K) Plb versus 5b mice (*P* < 0.01) (Figure 5, A and E; multimer-to-monomer quantification in Supplemental Figure 2C). No difference in the αS T:M ratio was detected between Plb Ctl and L444P-LD5b and L444P-5b (2-way ANOVA, *P* > 0.05; Supplemental Figure 3F). To assess whether the increase in the αS T:M ratio was associated with changes in αS buffer solubility and serine 129 phosphorylation, sequential extraction of cortical (Figure 5B) and midbrain (Figure 5C) homogenates was performed. While no differences in the relative TBS/RIPA-buffer solubility were detected between groups (Figure 5, F and H), both L444P and E326K Gba1 Plb mice showed a significant increase in buffer-insoluble pS129 in cortical and midbrain extracts (Figure 5, G and I). These increases in pS129 positivity were not observed after 5b treatment (Figure 5I). Testing for 5b treatment effects in Gba1 (L444P+E326K, combined) mice showed a significant decrease in pS129/total αS in brain cortex (*P* < 0.05, Figure 5G). In addition, dosing of 5b stepwise reduced the total pS129 level (Supplemental Figure 3D), and no differences in pS129/total αS were observed between Ctl, L444P-LD-5b, and L444P-5b mice (Supplemental Figure 3E). Together, the data suggest that 5b normalized GCase maturation and improved αS homeostasis in Gba1 mutant mice.

### SCD inhibition reduces pS129^+^ inclusions, normalizing lysosomal clustering and biogenesis in Gba1 mutant mice.

**Figure 6:**
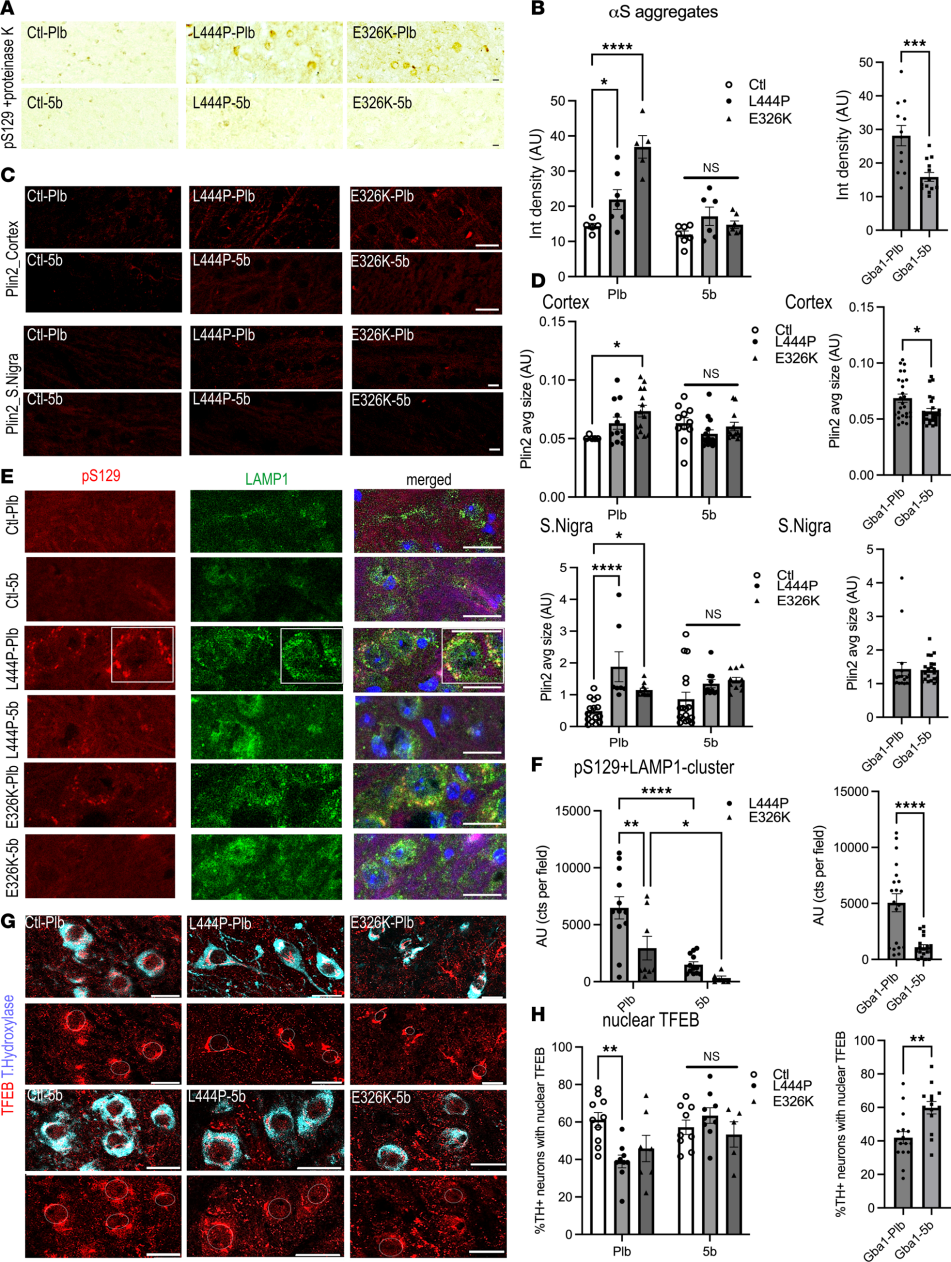
5b treatment reduces PK-resistant and vesicle/lipid-rich αS aggregates and normalizes lysosomal clustering and biogenesis. (**A** and **B**) Representative images of PK-resistant pS129^+^ aggregates in the cortex of Plb- and 5b-treated Gba1 mice and quantification (*n* = 2–3 sections, *n* = 3–4 mice per cohort). Note only background staining was seen in Ctl mice independent of treatment. (**C** and **D**) Confocal microscopy of cortical and midbrain (S. Nigra) region labeled with Plin2 (red) and quantification of puncta sizes in cortex and S. Nigra. (**E** and **F**) Midbrain sections triple labeled with pS129 (red), LAMP1 (green), and DAPI (blue) and quantification of LAMP1+pS129 clusters. (**G** and **H**) Adjacent sections were additionally stained for lysosomal biogenesis marker TFEB and tyrosine hydroxylase and graphs quantify the relative proportion of dopaminergic neurons displaying nuclear TFEB immunolabeling. Data are shown as mean ± SEM. Two-way ANOVA, Tukey’s post hoc test (**B**, **D**, **F**, and **H**, left panels). Two-tailed, unpaired 2-tailed *t* test comparing in Gba1 (E326K+L444P) Plb versus 5b (**B**, **D**, **F**, and **H**, right panels). *P < 0.05, **P < 0.01, ***P < 0.001; ****P < 0.0001. Scale bars: 25 μm.

Abnormal, membrane-lipid rich aggregates are found in human PD brain (26), in GBA1-PD patient-derived neurons (50), and in PD-type fly models (64). We consistently observed pS129^+^ multilaminar membranes and LB-type aggregates in 3K αS mutant mice with PD-like phenotypes (16). To address neuropathological characteristics of pS129^+^ deposits in Gba1 mutant mice, we searched for PK-resistant αS aggregates in cryostat sections. We applied a monoclonal antibody specific for pS129 and then digested the sections with PK (Figure 6A). Four months of 5b treatment efficiently decreased the build-up of larger-sized PK-resistant pS129 αS granules in Gba1 mutant mice (Figure 6A). The granular patterns of PK-resistant pS129 αS forms were relatively strong in neuronal somata of the cortical layers V and VI in L444P and E326K sections; therefore, these were used for quantification. Only very few and small pS129^+^ puncta were detected in Ctl mice, and these were similar to 5b-treated Gba1 mutant mice (2-way ANOVA, genotype × treatment; *P* = 0.0001) (Figure 6B). Testing for 5b effects in Gba1 (L444P+E326K, combined) mice showed a significant decrease in PK-resistant immunoreactive puncta in Plb versus 5b (*P* < 0.001; Figure 6B).

Since αS also acquires PK resistance when aggregating into LB-type lesions that can include lysosomes, LDs, and other lipid-rich vesicle membranes (26, 65), we next stained sections with the LD membrane marker perilipin 2 (Plin2) (Figure 6C). Notably, Plins have high affinity for membrane lipid packing defects (66), and the Plin2 affinity toward LDs covered with MUFA C18:1 is higher than with saturated FA (67). The Plin^+^ LDs are shuttled to the lysosome, where Plins are extracted (e.g., by cathepsin B) from LD membranes, enabling lipophagy of the LD content (68, 69). When quantifying the relative Plin2 cluster sizes in cortical (frontal cortex, layers V–VI) and midbrain (DAergic) neurons, a significant increase was detected in midbrain of both Plb L444P and E326K Gba1 versus Ctl, and these Plin2^+^ puncta were normalized after 5b treatment (Figure 6D; 2-way ANOVA, genotype × treatment, *P* < 0.05; for full-sized images, see Supplemental Figure 4. When quantifying the 5b treatment effects in Gba1 (E326K+L444P, combined) mice, we detected a significant decrease in Plin2^+^ puncta between Plb and 5b in cortex (*P* < 0.05; Figure 6D). We previously observed enlarged LAMP1+pS129 clusters in the DAergic neurons of 3K αS mice that were reduced by 5b treatment (45). The LAMP1+pS129 clusters were increased by Gba1 mutation and normalized by 5b treatment in Gba1 mutant 5b versus Plb across genotypes (Figure 6, E and F). Ctl mice only showed background staining when using the pS129 antibody (Supplemental Figure 5) and, therefore, were excluded from this analysis.

Nuclear TFEB is considered the master regulator of autophagy and lysosomal function and augments the expression of autophagy-lysosomal genes, thereby contributing to the degradation of cytoplasmic lipidic material (70–72). Time-laps experiments have shown that, under normal (fed) conditions, TFEB continuously shuttles between the cytosol and the nucleus (73). We investigated whether lipidic aggregates in Gba1 mutant mice associate with a change in the cytosolic/nuclear TFEB immunoreactivity in midbrain DAergic neurons (Figure 6G). Two-way ANOVA revealed a significant interaction (genotype × treatment; *P* < 0.01) with similar nuclear reactivity in 5b-treated mice (Figure 6H). Analyzing the 5b-treatment effect on Gba1 (L444P+E326K) showed that 5b significantly raised the level of nuclear TFEB (*P* < 0.01) (Figure 6H). Together, these data suggest that 5b treatment normalized the lipid-rich, pS129^+^ lysosomal aggregates and promoted lysosomal biogenesis and distribution in Gba1 mutant mice.

### 5b treatment decreases lipid unsaturation, normalizing phospholipid membrane homeostasis in Gba1 L444P and E326K mouse brain.

**Figure 7:**
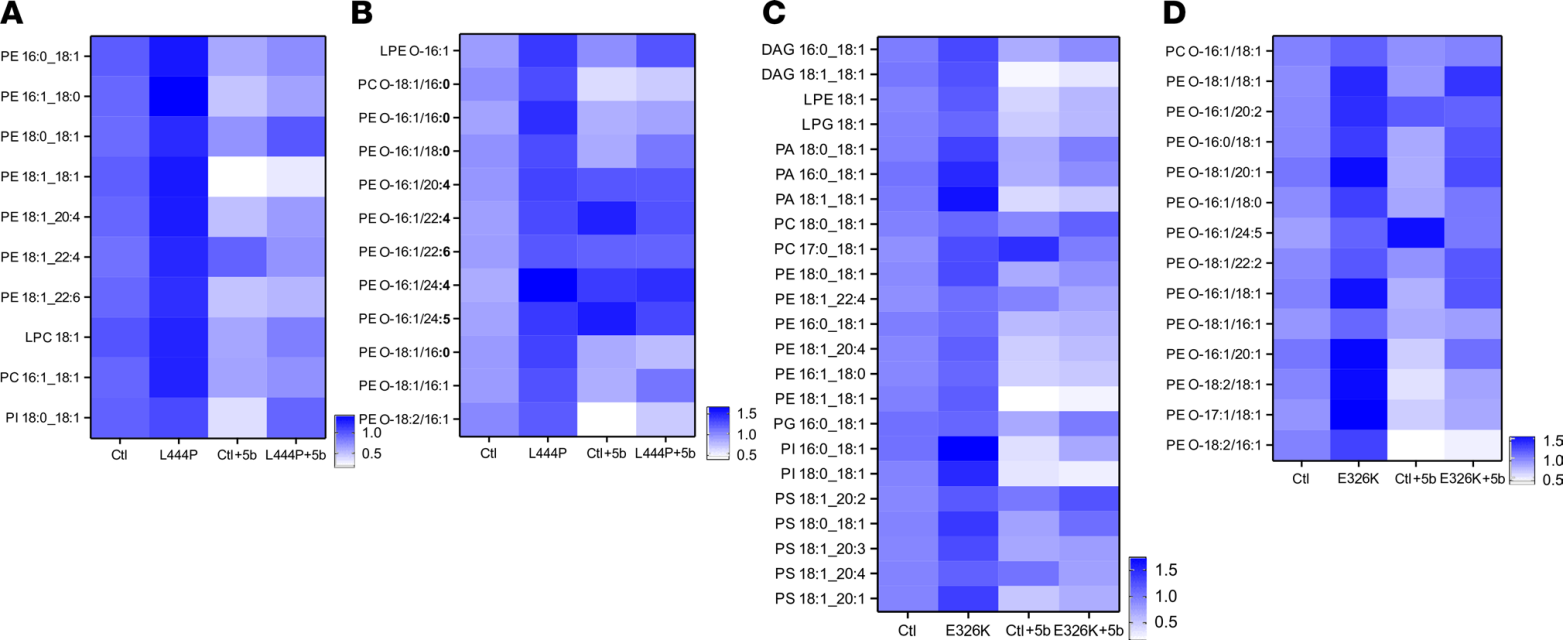
SCD inhibition (5b) treatment decreases lipid unsaturation in Gba1 mutant mice. Lipidome data (pmol) were analyzed with a focus on lipids meeting the following criteria: inclusion of C16:1 or C18:1 fatty acids and a minimum of *n* = 3 replicates. Lipid species with an average increased of > 20% in mice with a GBA mutation and decreased by > 20% by 5b treatment were selected for heatmaps. (**A**) Heatmap shows C16:1- and C18:1-containing phospholipid species (pmol) increased in GBA L444P mice relative to control mice with > 20% decrease upon 5b treatment. Heatmaps depict median values with each plot representing a minimum *n* = 3 per condition. (**B**) Data per **A** but showing ether lipids. (**C**) Data per **A** for Gba1 E326K mice versus controls. Two DAG species are shown in addition to phospholipids. (**D**) Data per **B** showing ether lipids.

To determine the specific lipids in which the C16:1 and C18:1 species were reduced, we performed a focused species and subspecies analysis of the mouse brain lipidome of the 16-month-old Plb- and 5b-treated mice (Figure 7). We first focused on C16:1 and C18:1 containing phospholipids with an average increase of > 20% in Plb L444P mice relative to Ctl and further selected for those decreased by > 20% following 5b treatment. As expected, 5b treatment reduced several C18:1- and C16:1-containing lipids, predominantly PE species, as well as PC, PI and LPC species in L444P mice (Figure 7A). There was a striking change in these monounsaturated species in ether lipids including phosphatidylethanolamine-o (PE-O) as well as phosphatidylcholine-o (PC-O) and lysophosphatidylethanolamine-o (LPE-O) (Figure 7B). The same analysis was performed comparing E326K versus Ctl mice with or without 5b treatment, and this resulted in the identification of a larger set of phospholipids being identified as increased by the Gba1 mutation and reduced by 5b treatment. The phospholipid headgroups were PE, phosphatidic acid (PA), PC, and phosphatidylserine (PS), among others (Figure 7C). Notably, there was enrichment of these MUFAs in diacylglycerides, and this was also reduced by 5b treatment (Figure 7C). Similar to our observations in mice with the L444P mutation, mice with the E326K mutation also had an increase in PE ether lipid species, and a subset of these was reduced by 5b treatment including many in the PE-O class (Figure 7D).

## Discussion

Here, we assessed whether a brain penetrant SCD inhibitor, 5b, can prevent the PD-like neuropathology in GBA1 mutant neuronal culture and in 2 Gba1 mutant mouse lines, expressing either the more common and severe L444P mutation or the E326K mutation—both risk factors for the development of PD (74, 75). Fatty acid and lipid profiling revealed an increase in SCD-produced unsaturated fats, in particularly evident in the C16 FADI, in both GBA1-PD culture and in mouse brain. The increases in MUFAs were coupled with an increase in *SCD1* transcriptional level. Such an increase was associated with neurodegeneration, as was recently reported in the brain of an AD mouse model (76), which may also benefit from SCD inhibition (42, 43). Our assessment of motor phenotypes showed impaired motor performances in 12-month-old Gba1 (L444P and E326K) mutant mice. This differs from previous studies reporting motor impairments only in mice aged 24 months (E326K) (53) or no phenotypes in L444P tested once at age 16 months (62). The difference in the observed phenotypes might be due to a more stringent protocol (rotarod training, number of testing days, and trials) and the overall better performance of control mice, having been trained already at a younger age and thus able to perform close to the maximum *rpm* (~270- to 300-second endurance) (16, 77). In addition, we found a decrease in the brain αS T:M ratio, GCase maturation, and activity and an increase in insoluble pS129^+^ underlying the motor phenotypes at 12 months. A previous study of L444P mouse primary cortical neurons reported a decrease in the αS T:M ratio (23); in our current study, we validate this finding in dissected cortices of L444P mice and in human L444P PD patient-derived neurons. While we found more of the DAergic pathologies in aged L444P mice (16 months; e.g. dopamine level, Plin2^+^ LD clusters, and cytosolic TFEB), some of the cortical pathologies were more strongly detected in E326K Gba1 mice: cortical PK-resistant αS aggregates and Plin2^+^ LD clusters. These pathologies may contribute to the more severe rotarod deficits observed in the E326K mice, since frontal and motor cortices are heavily involved in learning of this motorically challenging task (78).

We report that daily oral administration of a well-characterized SCD inhibitor (46) markedly prevents these phenotypes. Pharmacologically decreasing the SCD enzymatic activity in Gba1 mice improved the more severe neuropathology, including PK-resistant αS aggregation and the rotarod deficit. In cultured GBA1 mutant neurons, we observed a 5b-mediated increase in the αS T:M ratio, similar to the increase we observe in Gba1 mutant mouse brains. In addition to the more physiological αS homeostasis, we observed an increase in TFEB (lysosomal biogenesis), lysosomal distribution, and normalization of the mature GCase level, which may all contribute to healthy lysosomal function. These findings, which demonstrate the first report to our knowledge of the ability of SCD to regulate brain WT αS homeostasis in Gba1 mutant mouse brain and patient neurons, highlight the upstream function of physiological αS tetramers and FA metabolism as modulators of αS in vivo.

Our results support the hypothesis that decreasing the relative level of MUFAs protects against a PD-like syndrome in GBA-mutant mice, consistent with previously demonstrated effects of unsaturated fats contributing to pathological αS oligomerization in cultured neurons (44, 79, 80). Other studies have reported that supplementation with saturated FA can stimulate neuroprotection in human PD patient neurons (44) or in PARKIN and PINK mutant PD-type fly models (81, 82). In addition, intragastric gavage of 8:0 SFA has been associated with improved DA signaling in a MPTP acute PD mouse model (83). With direct clinical relevance to DAergic neurodegeneration in PD, we observe an increase in striatal DA levels and TH^+^ nerve terminals coupled with motor improvements in Gba1 mutant mice undergoing prolonged SCD inhibitor treatment.

Here we show that an increase in the FA saturation state can increase the physiological αS T:M ratio and mitigate the neuropathological Gba1 mutant phenotype. Emerging evidence suggests that αS monomers have an affinity for highly curved phospholipid membranes (84, 85), as consistently observed by αS overexpression across species (86–90). Therefore, a mechanistic explanation for our therapeutic benefit of shifting the FA balance toward the saturated state is the direct stabilization of the αS amphipathic helix that is induced upon its binding to saturated membranes (85). Notably, adding 16:0 SFA in the form of lyso-PC micelles to pure WT αS in vitro has been reported to directly stabilize the α-helical conformation of human WT αS protein (hu WT αS) (91). In addition, the αS N-terminus harbors a motif homologous to a region in fatty acid–binding proteins (92), which may thus facilitate the interaction of αS with FA on certain membrane lipids. Thus, in Gba1-5b–treated mice, the relative increase in 16:0 phospholipids by SCD inhibition may enhance physiological αS tetramerization and the transient nature of αS vesicle binding, lessening the pathogenic consequences of free αS monomers.

Interestingly, histological analyses have revealed prominent lipid-rich membranes in brainstem (93) and cortical LBs (94), but their derivation remains unknown. Lipid dyshomeostasis is increasingly observed to cooccur with αS aggregation (recently reviewed ref. 37), and several reports now demonstrate how manipulations that alter lipid levels promote αS aggregate formation in vitro (91), in yeast (34), and in rodent and human neuronal cultures (44, 55). We found the LD membrane marker Plin2 accumulating in brain regions with αS pathologies of GBA mutant mouse brain. Plin2 is an enzyme inserted into LD membranes with packing defects and with high affinity toward unsaturated PC-covered (18:1/18:1) LDs but low affinities for saturated PC-covered LD (67). The observed upregulation of unsaturated fats (PC, PE, and others) by Gba1 mutations may explain the accumulation of Plin2^+^ LDs and their coaggregation with αS at lysosomes. Plins protect against cytosolic lipolysis but are stripped off by lysosomal lipases (such as cathepsin B; ref. 69), enabling autophagic turnover of the lipid content (68). Interestingly, variants in cathepsin B are an additional risk factor for PD penetrance in human mutant GBA1 (hu GBA) carriers (95). Thus, the accumulation of Plin2^+^ LD markers is further evidence for increased FA membrane unsaturation and impaired lipophagy with Gba1 mutations; it also indicates that SCD inhibition acts against these pathologies. Additionally, an increase, αS level has been shown to promote GCase dysfunction (3) and also to correlate with an increase in unsaturated membrane lipids, and in particular PEs, associating with increased lipid-peroxidation and vulnerability of DAergic neurons (41, 96). This may explain the increase in PE and PE-O we find in Gba1 L444P and E326K mice, which also displayed more (soluble and RIPA-insoluble) αS versus Ctl mice.

Together, our previous and present findings highlight the effect of reestablishing the normal tetramer levels consistent with compelling evidence that the lipid homeostasis is a major contributor of a physiological αS T:M ratio. Our study provides the strong preclinical animal data to advance this disease-modifying approach to testing in PD patients with GBA1 mutations.

## Methods

Supplemental Methods are available online with this article.

### Sex as a biological variable.

Only male mice were included in this study in order to reduce variability by using a homogenous experimental population and thereby reducing the total numbers of mice required, in accordance with NC3R guidelines.

### GBA1 patient-derived iPSC neurons.

GBA1 mutant L444P and isogenic correction cells were provided by the M. Deleidi (Imagine Institute of Genetic Diseases) and were previously described (50). GBA mutant E326K cells were sourced from the National Institute of Neurological Disorders and Stroke (NINDS) Human Cell and Data repository. Corresponding neurogenin-expressing lines were generated by the BWH iPSC NeuroHub. Virus was made as previously described (97) with FUT-TetO-P2A-puromycin (Addgene plasmid no. 52047) and FUW-M2rtTA (Addgene plasmid no. 20342). iPSC lines were transfected at an MOI of 30 and expanded as feeder-free cells in defined, serum-free media (mTeSR, Stem Cell Technologies). NGN-2 iNs (neurons) were induced per previous protocols (98) with minor modifications as previously described (22).

### Cell culture and 5b treatment.

Was conducted as previously described (18). Further details can be found in the Supplemental Methods.

### Intact-cell crosslinking and analysis.

L444P mutant and corrected lines were plated at a density of 200,000 cells per well in a 24-well plate. E326K cells were plated at 250,000 cells per well. For crosslinking, cells were incubated with 0.5 mM of the cell-penetrant crosslinker DSG (Thermo Fisher Scientific, 20593) for half an hour at 37°C; excess DSG was neutralized with 1M Tris Hydrochloride (Thermo Fisher Scientific, BP1757-100). Cells were harvested and lysed in phosphate-buffered saline with 1% Triton X-100 containing 1:1,000 phosphatase and protease inhibitor (PPI; Thermo Fisher Scientific, A32959) on ice for 20 minutes, before being spun at 15,060*g* for 30 minutes at 4°C. Crosslinking efficiency was evaluated using a DJ-1 dimer:monomer ratio control. Equal crosslinking observed for the DJ-1 control blots was a criterium for data inclusion.

### Immunoblotting.

Cell lysates were prepared by the addition of NuPAGE LDS sample buffer and boiling for 4 minutes. Electrophoresis was conducted on NuPAGE 4%–12% Bis-Tris gels with NuPAGE MES-SDS running buffer. Gels were transferred to iBlot 2 PVDF Regular Stags (Invitrogen, IB24001) via the iBlot P0 protocol. Blots were fixed in 4% paraformaldehyde in PBS, blocked in I-Block (Thermo Fisher Scientific, T2015) in PBS containing 0.1% Tween-20 (PBS-T), and incubated overnight at 4°C in primary antibody (Syn1, BD610787, 1:1,000, BD Bioscience; DJ-1, AB76008, 1:5,000, Abcam) with agitation. Blots were washed 3 times in PBS-T for approximately 10 minutes/wash before the application of secondary antibody (1:5,000) in I-Block PBS-T for 45 minutes at room temperature (RT) with agitation. An additional wash step was conducted before developing the blots with the electrochemiluminescence agent (Promega, W1001). Bands were quantified using LI-COR Image Studio software.

### Mouse models.

E326K (a gift from Han Seok Ko, Johns Hopkins University, Baltimore, Maryland, USA) (60) and L444P (The Jackson Laboratory, 024574) (99) Gba1 mutant mice have been described and bred to homozygosity, and heterozygous breeding with C57BL/6 mice (Charles River) were used to create Ctl mice. Mice were housed on a 12-hour light-dark cycle with lights on at 7:00 a.m., RT at 21°C–23°C, and humidity at 55%–60% and had access to regular chow and water ad libitum. For 5b treatment, 12-month-old Ctl (C57BL/6), E326K and L444P GBA mutant mice were administered the compound accessible ad libitum in standard diet (Research Diets Inc, New Brunswick, NY).

### Intact-cell crosslinking of brain tissue.

Dissected brain regions were gently minced into small bits with a razor blade, and the brain bits were washed free of released cytosol and resuspended in PBS with EDTA-free Complete protease inhibitors (Roche Applied Science). Intact-cell crosslinking was then conducted on the washed brain bits as previously described (59) with minor modifications. Briefly, the cell-permeable crosslinker DSG was prepared at 1 mM final concentration in DMSO immediately before use. Samples were incubated with crosslinker for 40 minutes at 37°C with rotation. The reaction was quenched by adding Tris, pH 7.6, at 100 mM final concentration and incubated for 10 minutes at RT. After quenching and aspiration of the supernatant, proteins in intact tissue were extracted directly in TBS/1% Triton X-100.

### Sequential tissue extractions.

The regional expression pattern of αS was initially examined at age 12 months (prior treatment) in a subset of mice and then after treatment in cohorts at age 16 months. Mice were anesthetized and decapitated, and the brains were dissected on a chilled stage. Sequential extractions were performed as described (100). Briefly, tissues were homogenized in 2.5 volumes of TBS (50 mM Tris-HCl [pH 7.4], 175 mM NaCl; 5 mM EDTA; protease inhibitor cocktail; Calbiochem) and spun for 20 minutes at 120,000*g*. The pellet was subsequently extracted in TBS with the addition of 1% Triton X-100, then in TBS with the addition of 1M sucrose, The TX-insoluble pellet was then extracted in RIPA buffer (TBS, 1% NP-40, 0.5% sodium deoxycholate, 0.1% sodium dodecyl sulphate), with each extraction step followed by ultracentrifugation for 30 minutes at 120,000*g*.

### RNA.

RNA was measured as previously described (46). Briefly, total RNA samples were isolated from brain cortices using mirVana miRNA Isolation Kit (catalog AM1561), and RNA concentrations determined by Nanodrop. The samples were converted into cDNA using Applied Biosystems High-capacity cDNA reverse transcription kit (catalog 4368813). Afterward, the cDNA was mixed with TaqMan Fast Universal PCR Mix (catalog 4352042) for qPCR analysis using TaqMan Gene Expression primers SCD1 (Mm00772290) and GAPDH (Mm99999915_g1).

### WB analyses.

Total protein (8–15 μg) of sequential extracts of dissected mouse brain regions were electroblotted onto nitrocellulose membranes (MilliporeSigma). For improved immunodetection of αS (monomers of which are prone to washing off filters; ref. 101, 102), the membranes were fixed in 4% paraformaldehyde (PFA) for 10 minutes. After washing in phosphate-buffered saline (PBS), membranes were blocked for 1 hour at RT in PBST (phosphate-buffered saline with 0.2% Tween-20) containing 5% bovine serum albumin (BSA). Blots were then incubated with mouse GCase (G4171; MilliporeSigma; 1:1,000), mouse αS antibody (syn1, 1:1000; BD biosciences) or an antibody against phosphorylated (ser129) αS (51253; Abcam; 1:5,000) or in PBST containing 5% BSA overnight. After washing with PBST, membranes were probed with appropriate secondary antibodies (American Qualex, 1:5,000), visualized with enhanced chemiluminescence (ECL, PerkinElmer), and analyzed with the VersaDoc gel imaging system. Proteins were normalized to b-actin (A5441, MilliporeSigma; 1:3,000) used as a loading control. DJ-1 (ab76008, Abcam; 1:2,000) was used as a control for crosslinking and loading. Quantification of signal intensities was performed as described (103). Blots shown in Figure 3E and Figure 5, B and C, quantifying monomeric aS were detected with established (>300 references each) antibodies specific for total αS, pS129, or actin (see manufacturer information; Abcam and MilliporeSigma).

GCase activity assay was conducted as previously described for cell culture (104) and mouse brain (ab273339, Abcam) and details for the measurement can be found in the supplemental information.

### High pressure LC (HPLC).

HPLC was conducted as previously described (105). Details for the measurement can be found in the Supplemental Methods.

### IHC.

Mice were sacrificed with an overdose of isoflurane, followed by intracardiac perfusion with PBS and ice-cold 4% (w/v) PFA in PBS (pH 7.4). The brain was dissected from the skull and post-fixed in 4% PFA for another 48 hours at 4°C. Brains were cut into 25 μm cryotome sections, and double-labeling was performed as described (100). Briefly, sections were blocked in 10% normal goat serum and incubated overnight at 4°C with antibodies to anti pS129 αS (51253; 1:2,000; Abcam), rabbit anti-TH (AB 152, 1:500; MilliporeSigma), or chicken anti-TH (AB76442; 1:500; Abcam), Plin2 (A6276; 1:200; ABclonal), or TFEB (A700-070; 1:200; Bethyl-Laboratories). This was followed by incubation with the appropriate FITC-conjugated secondary antibodies (1:250 in PBS; Alexa Fluor 488, 568, and 647) for 3 hours at RT. Nile Red was diluted 1:500,000 in staining buffer (Nile Red Kit; N1142, Invitrogen), applied for 15 minutes, washed 5 times for each 5 minutes in PBS at the final staining step, and then embedded with DAPI-containing mounting medium (Vectashield).

Details for confocal microscopy and Image J analysis are included in the Supplemental Methods.

### Assessment of striatal DA fiber integrity.

Assessment of striatal DA fiber integrity was performed as previously described and details can be found in the Supplemental Methods.

### Behavioral testing.

All behavioral testing was conducted as previously described (16, 77).

### Gait scan.

Automated gait analysis was performed using Treadscan (Cleversys Inc.). Gait patterns of 3- to 6-month-old mice were measured for 20 seconds at a speed of 13 cm/sec on a transparent running belt illuminated by a LED light and reflecting footprints captured by a video camera positioned underneath the walkway.

### Rotarod.

Motor coordination and motor skill learning were evaluated using an accelerating rotarod (Ugo Basile), and time spent on the rod was recorded. The first 2d consisted of a habituation trial at constant speed (4 rpm for 5 minutes), followed by 2 trials of 440 rpm progressive acceleration within 5 minutes followed by 3d with accelerating trials (4–40 rpm, 5 minutes). An intertrial pause of at least 1 hour was applied to avoid fatigue and stress, and a maximum cutoff of 5 minutes was used.

### Lipid sample preparation, profiling and analysis (Lipotype and OmegaQuant).

Lipid profiling of tissue samples was performed as previously described (22), and their details can be found in the Supplemental Methods.

### Statistics.

Details regarding each statistical test, biological sample size (*n*) and *P* value can be found in the corresponding figure legends. All data are represented as mean ± SEM of independent experiments. In all experiments, the genotypes can be found in the corresponding legends. Data were collected and processed side by side in randomized order for all experiments; most analyses were routinely performed blind to the conditions of the experiments. Unpaired, 2-tailed *t* tests were used for comparison between 2 groups, with *P* < 0.05 considered significant. For all comparisons involving multiple variables, a 1- or 2-way ANOVA was performed followed by the appropriate post hoc test for multiple comparisons using *P* < 0.05 for significance. An average abundance heatmap for each cohort is provided to highlight the more abundant FAs, and data were examined by standard principal component analysis (Lipotype, Dresden). For all experiments, between 4 and 6 (biochemistry, histology, lipid profiling) and betweem 4 and9 (behavior) mice were used. All statistical analyses were preformed using GraphPad Prism 10 software.

### Study approval.

All animal experiments were performed in accordance with the *Guide for the Care and Use of Laboratory Animals* (National Academies Press, 2011) and were approved by the Animal Ethics Committee of the Brigham and Women’s Hospital (approval no. 2016N000314). The methods used for the supplemental figures are described in the Supplemental Methods. Information for reagents and antibodies, and other materials used are described in the Supplemental Methods.

### Data availability.

Values for all data points in graphs are reported in the Supporting Data Values file. Any additional underlying data can be obtained from the corresponding author upon request.

## Author contributions

SN was responsible for the overall direction of the mouse project and SF of the human iPSC neurons. HH and SF were responsible for Figure 1, Figure 4A, Figure 7, and Supplemental Figure 1; EK was responsible for WB analysis of Figures 3 and 5; TEM was responsible for Figure 6G; SJR was responsible for Supplemental Figure 2A; AT and GPHH were responsible for Supplemental Figure 1C; ES was responsible for Figure 6, C and E; WH and RP were responsible for Figure 2, A and B, and Figure 3B; MAA was responsible for Figure 4D and Figure 6A; MB was responsible for Figure 3D and Supplemental Figure 2D; XZ was responsible for Figure 4F; SK, HSK, PS, and DJS provided expert advice of the manuscript. SN, HH, TEM, SJR, GPHH, and SF contributed to the conception and design of the study. All authors contributed to the acquisition and analysis of the data. SN, HH, AT, GPHH, TEM, XZ, and SF contributed to drafting of the text and preparation of the figures. All authors contributed to editing the manuscript and approved the final draft.

## Supplementary Material

Supplemental data

Unedited blot and gel images

Supporting data values

## Version 1 06/03/2025

In-Press Preview

## Version 2 07/08/2025

Electronic publication
