# Supplementary material for: Stearoyl-CoA desaturase inhibition normalizes brain lipid saturation, α-synuclein homeostasis, and motor function in mutant Gba1-Parkinson mice
Source: JCI Insight. 2025 Jun 3;10(13):e188413. doi: 10.1172/jci.insight.188413 (PMC12288896; doi:10.1172/jci.insight.188413)
Supplement: Supplemental data [file jciinsight-10-188413-s191.pdf]

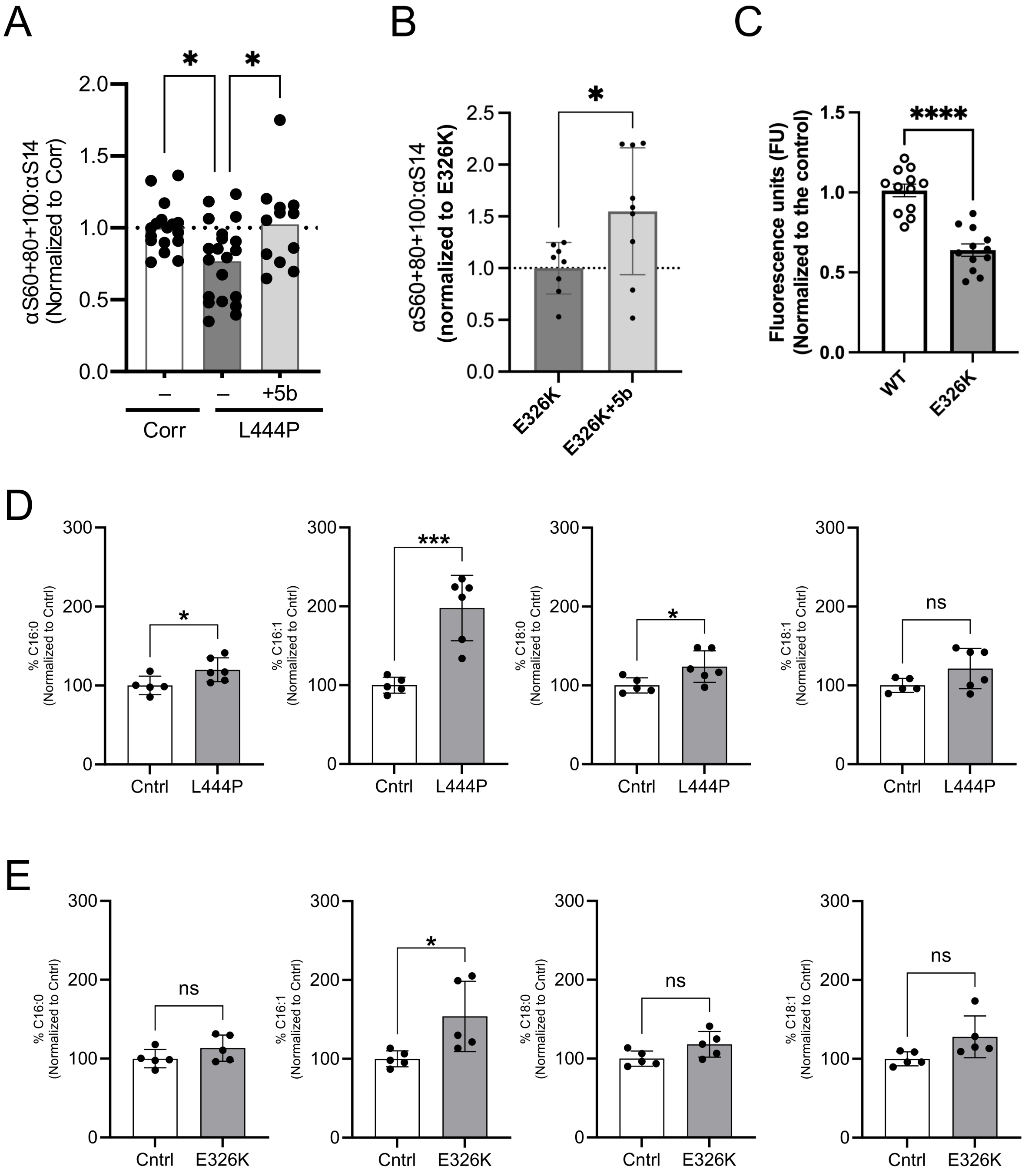

Supplemental Figure 1.

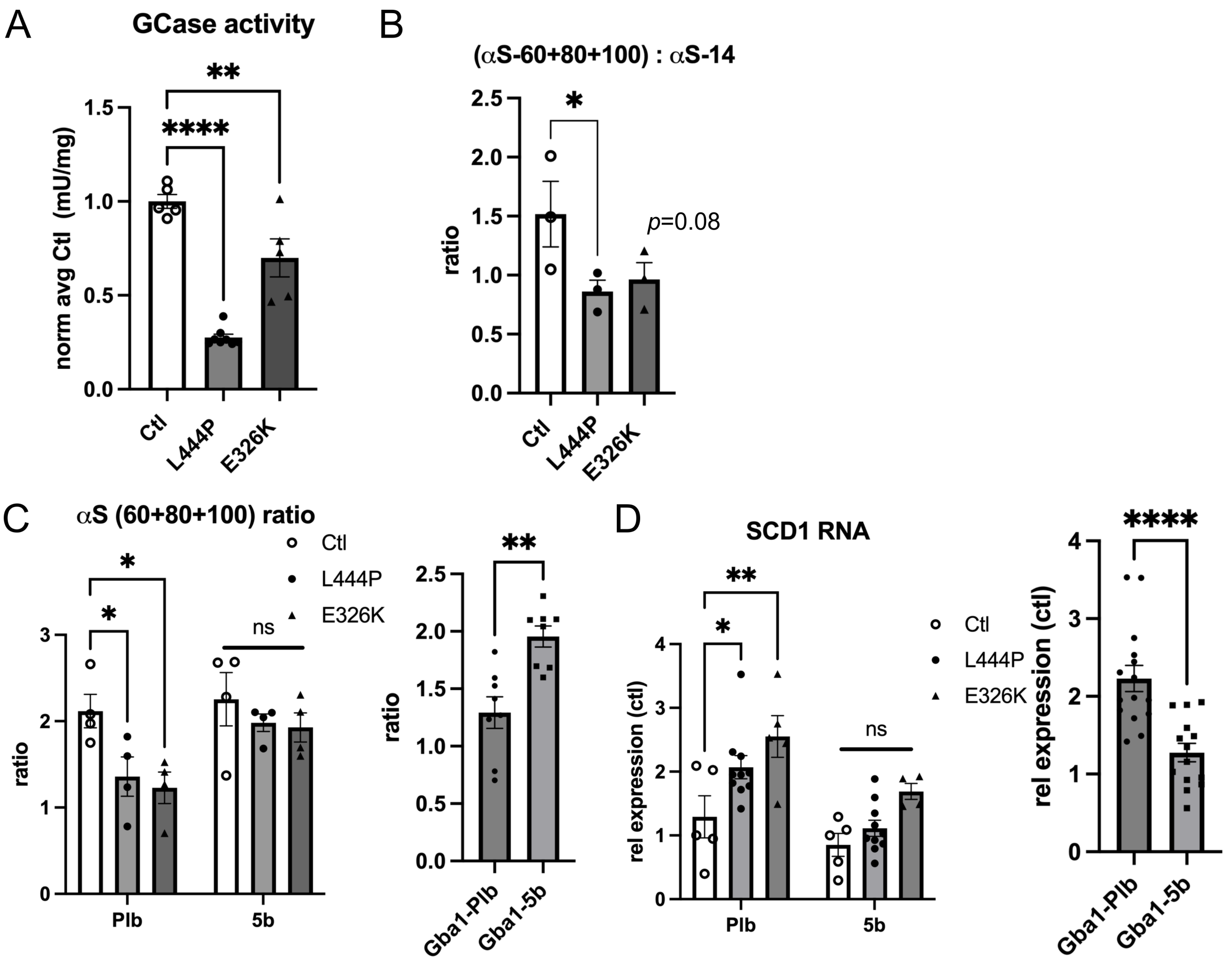

Supplemental Figure 2.

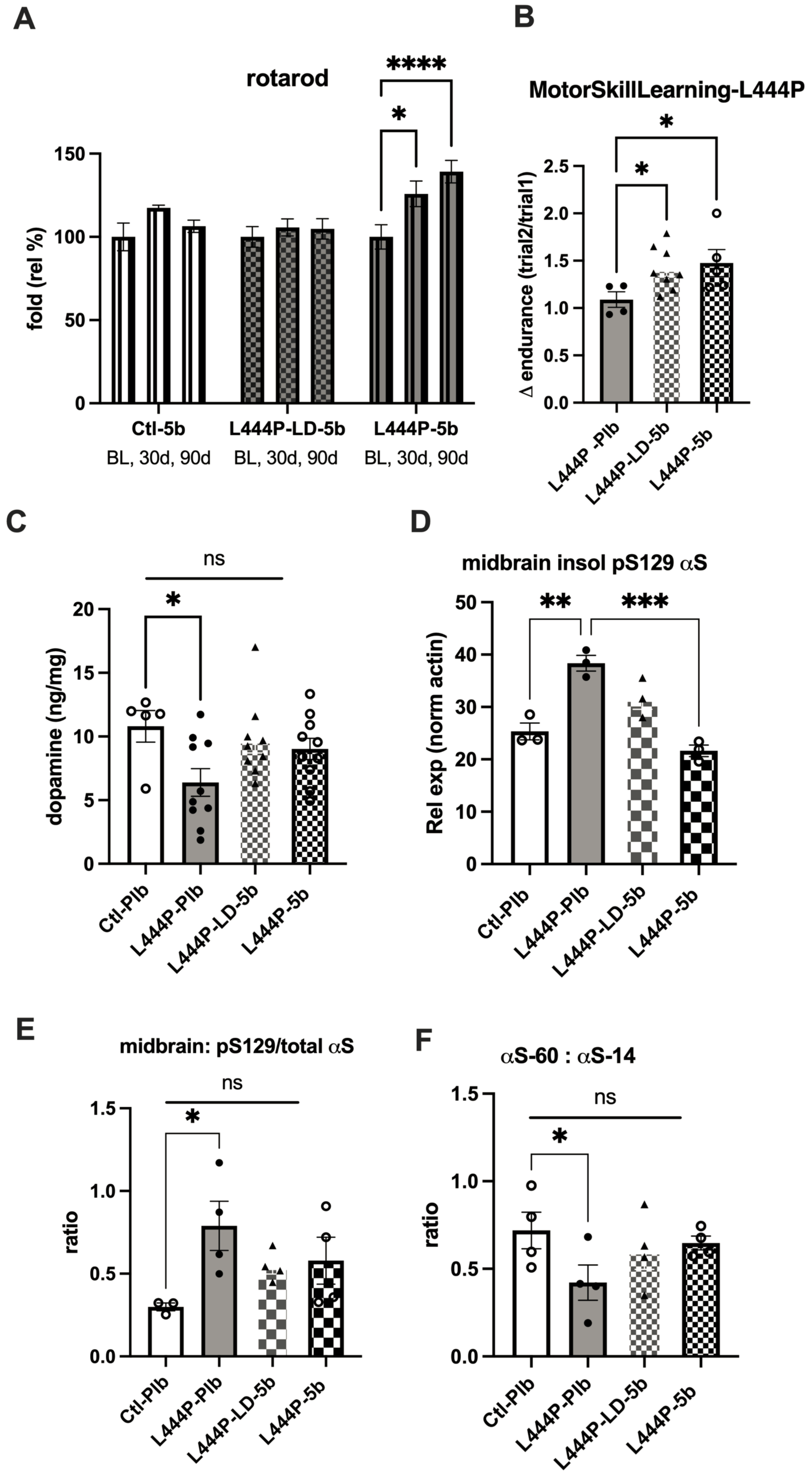

Supplemental Figure 3.

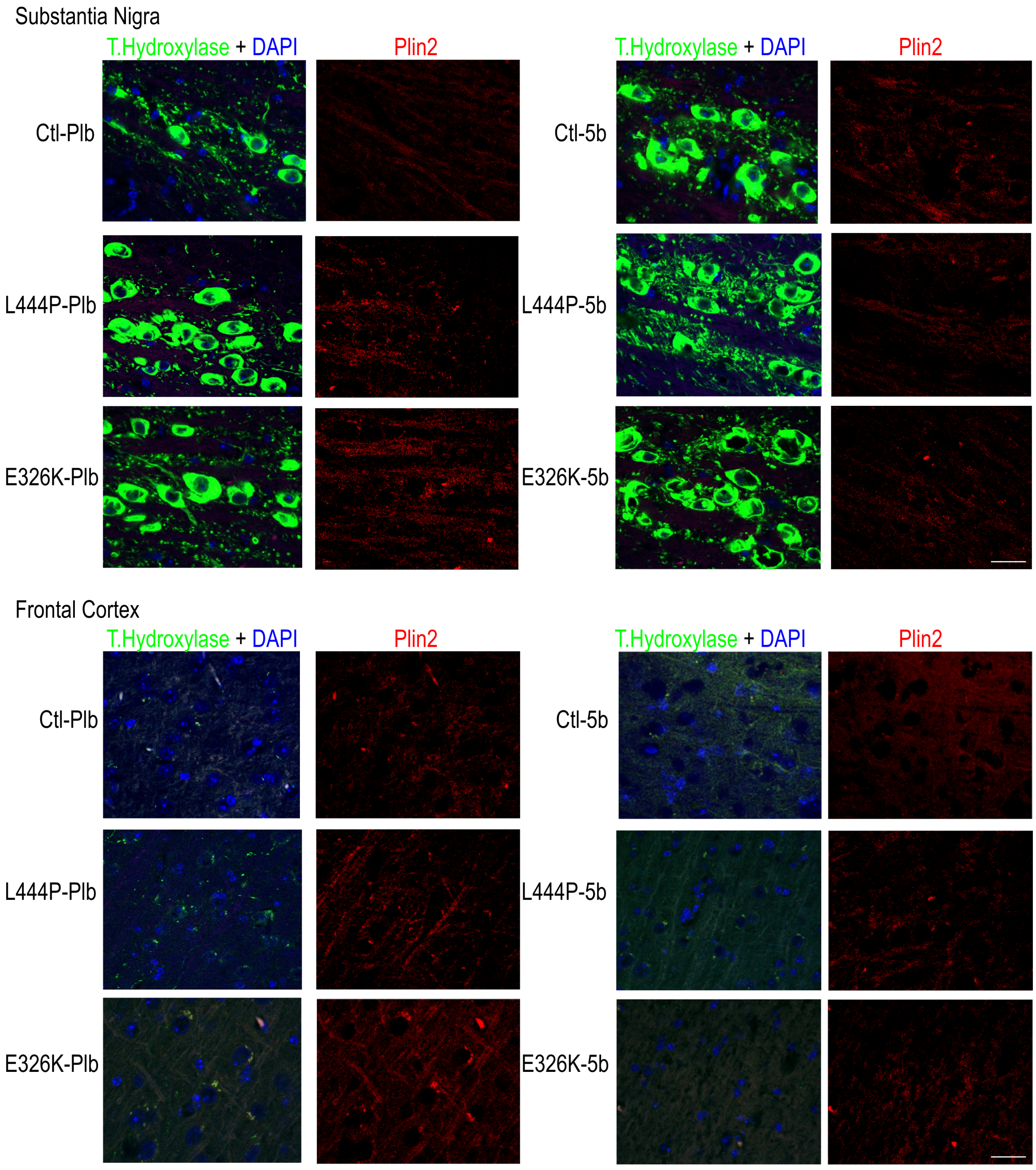

Supplemental Figure 4.

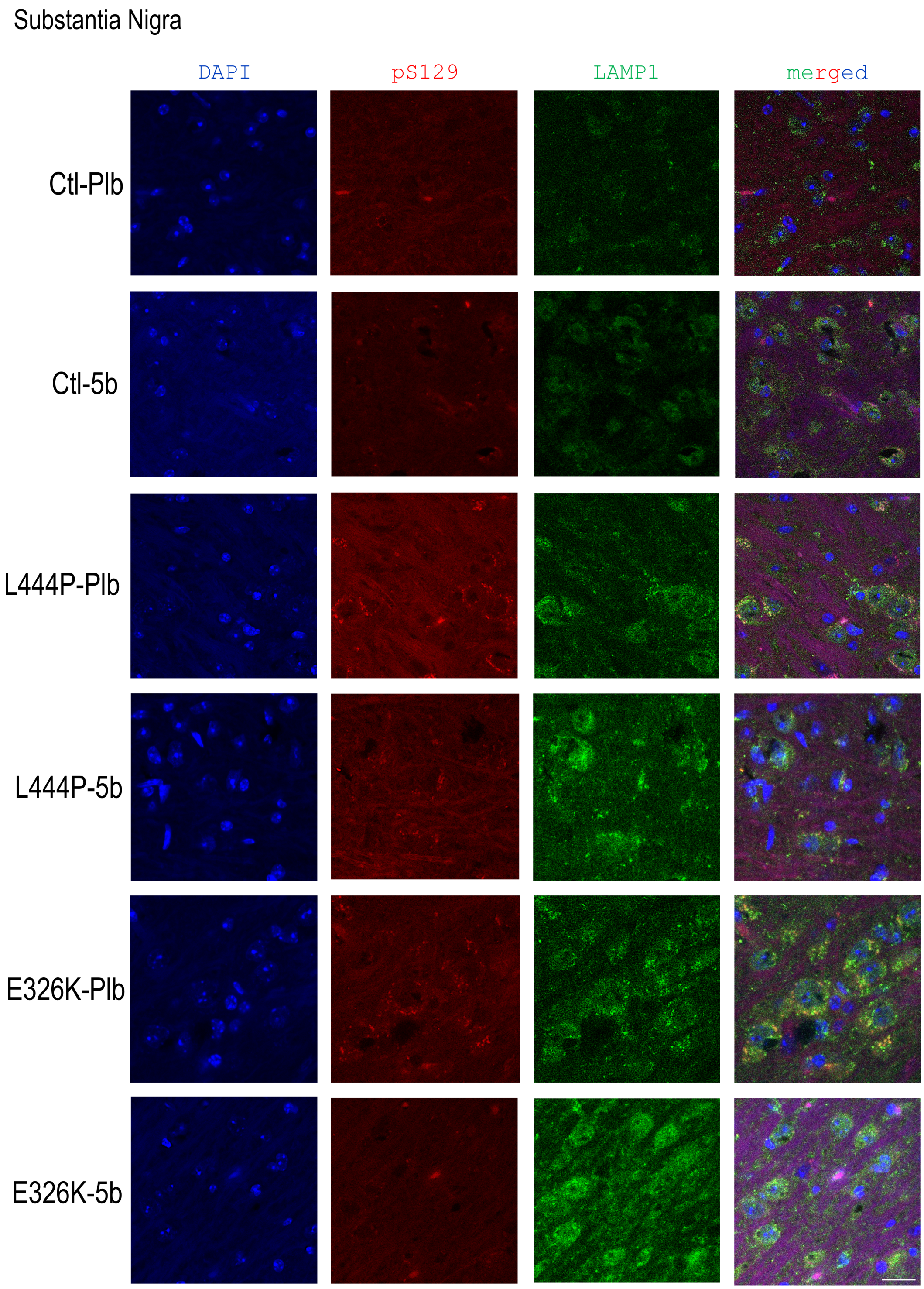

Supplemental Figure 5.

SUPPLEMENTAL FIGURE 1. SCD inhibition normalizes αS multimer/monomer ratio of patient-derived GBA1 L444P and raises the level in E326K mutant iPSC neurons.

(**A**) **L444P**: Quantification of the multimer-to-monomer ratio (αS60+80+100):αS14. Two-way ANOVA: p=0.011), statistically significant effects of condition ($F_{1,46}$ = 14.10, p=0.0066) and treatment ($F_{1,46}$=14.04 p=0.0067), Tukey's HSD test, Corr–5b vs. L444P–5b, p = 0.0323, L444P–5b vs. L444P+5b, p = 0.0328; N = 2, n = 12–19). (**B**) **E326K:** Quantification of αS60+80+100):αS14 ratio (p=0.32; two-tailed unpaired *t*-test; N = 1, n = 8–9). **(C)** GCase activity measured in DIV 18-22 cortical neurons of GBA1-E326K iPSC using the 4MUG assay. **(D), (E)** Total cellular C16:0, C16:1, C18:0 and C18:1 of L444P and E326K Gba1 mouse cortices were measured by gas chromatography. Data is reported relative to age-matched control (C57Bl6) mice. Statistical analysis: GraphPad Prism 10, two-tailed unpaired *t*-test.

SUPPLEMENTAL FIGURE 2. **GCase activity, SCD expression and αS multimerization in Gba1 mutant mice.** (**A**) Graph quantifies GCase activity (abcam; ab273339/K2003-100) in 12 mos Gba1L444P and E326K vs. ctl mouse cortices. (**B**) Graph quantifies αS (60+80+100):14 kDa ratio from blots such as shown in *Figure 3.* (**C**) Graph quantifies αS (60+80+100):14 kDa (T:M) ratio from blots such as shown in *Figure 5.* (**D**) Graph quantifies relative SCD1 RNA expression level relative to Ctl in Plb and 5b. For context of our hypothesis a part of this data is also shown in Fig. 3C. Data are mean ± SEM. One-way ANOVA post Tukey. *p<0.05, **p <0.01, ***p<0.001, ****p<0.0001.

SUPPLEMENTAL FIGURE 3. **Treatment with 7.5 mg/kg (low dose) 5b normalizes dopamine level, αS homeostasis and improves rotarod motor skill learning in Gba1 L444P mice.** (**A**) Graph quantifies balancing motor skill learning on a 4-40 rpm accelerating rotarod. (**B**) Significant rotarod improvement between the 2nd and 1st trial (motor skill learning) on the first testing day in L444P treated (LD-5b or 5b) vs. Plb L444P. (**C**) HPLC assay of striatal dopamine measured by HPLC shows relative similar level between 5b treated and Plb Ctl and significantly reduced DA level in L444P-Plb. (**D**), (**E**) Quantified from blots such as shown in *Figure 5* for pS129 and pS129/total αS in buffer insoluble (RIPA) extracts of the midbrain. (**F**) Graph quantifies αS 60:14 kDa (T:M) ratio from blots such as shown in Figure 5A. Data are mean ± SEM. One-way ANOVA post Tukey. *p<0.05, **p <0.01, ***p<0.001, ****p<0.0001.

SUPPLEMENTAL FIGURE 4.

Full sized confocal microscopy images of cortical and midbrain (S.Nigra) region labeled with Plin2 (red) and T.Hydroxylase (green) shown in *Figure 6*. Scale bar = 25 μm.

SUPPLEMENTAL FIGURE 5.

Full sized confocal microscopy images of sections triple labeled with pS129 (red), LAMP1 (green), DAPI (blue) shown *in Figure 6*. Scale bar = 25 μm.

SUPPLEMENTAL MATERIAL AND METHODS

Cell culture and 5b treatment

D4 iNs were thawed in thawing media and plated in plating media. Plates were coated with matrigel matrix basement membrane (Corning 354234) at 34.8 µg/cm^2 and incubated overnight at 37°C a day prior to plating; matrigel was aspirated immediately before plating. A full media change using D5 media was conducted the day after plating (D5). Half media changes were conducted on D9 and D16 using maintenance media incorporating 5b drug addition. Cells were treated with 1 µM of the SCD inhibitor 5b or a proportional amount of DMSO on D9, D16, and two days prior to harvest.

Neurobasal complete media (NBM complete) was prepared from pure NBM (Gibco 21103) with the addition of 20% dextrose, GlutaMAX (Gibco 35050), and MEM NEAA (Invitrogen 11140-050) to yield respective final concentrations of 0.3%, 2 mM, and 1x, then sterile-filtered and stored at 4°C. 10 µg/mL solutions of BDNF, CNTF, and GDNF (Peprotech 450-02, 450-13, and 450-10, respectively) were prepared by dissolving into 0.1% BSA in PBS. Y-27632 ROCK inhibitor (STEMCELL Technologies 72304) was dissolved in DMSO to make a 10 mM solution. Doxycycline hyclate (Sigma D9891) was prepared as a 20 mg/mL solution in sterile water. B27 (Gibco 17504044) and puromycin (Life Technologies A11138-03) were used at the manufacturer's concentrations. All media were prepared and equilibrated to 37°C prior to use as follows. Thawing Media: NBM complete with 10 µM Y-27632 ROCK inhibitor; Plating Media: NBM complete with 10 ng/mL BDNF, CTNF, and GDNF each, 10 µM Y-27632 ROCK inhibitor, 1x B27, 5 µg/mL puromycin, and 2 µg/mL docycyline hyclate; D5 Media: NBM complete with 10 ng/mL BDNF, CTNF, and GDNF each, 1x B27, 5 µg/mL puromycin, and 2 µg/mL docycyline hyclate; Maintenance Media: NBM complete with 10 ng/mL BDNF, CTNF, and GDNF each, 1x B27.

Cell culture- GCase enzymatic activity

At DIV 18-21, cells were lysed in the activity buffer (0.05M Citric Acid, 0.1 M K_2HPO_4, 0.01% BSA W/V,0.25% Triton v/v and 0.25% Taurocholic acid v/v, pH 4.5) supplemented with 1X protease inhibitor (Thermo Fisher) for 15mins on ice. The cell suspension was then briefly sonicated using Bioruptor (Diagenode) 5 cycles of 30s On/Off at low intensity. Protein quantification was performed using BCA kit (Pierce, Invitrogen). For the GCase enzymatic activity 5mM of 4-methylumbelliferyl β-D-glucopyranoside (4MUG) (Sigma) was incubated with 25µg of the total protein lysate in 1X activity buffer pH 4.5 for 30 mins at 37˚C in a

100µl reaction. The reaction was stopped with equi-volume of stop solution (1M glycine, pH 10.2). Fluorescence intensity (ex = 365 nm, em = 445 nm) was measured in a microplate reader (infinite M plex, Tecan) and normalized fluorescence units (FU) were plotted using graph pad prism v10.

Mouse brain - Glucocerebrosidase activity assay- mouse brain

The GCase enzyme activity was measured using the Glucosylceramidase Activity Assay Kit (Fluorometric, ab273339) according to the manufacturer's instructions, with small modifications. Cortical brain bits were homogenized with RIPA buffer and the pellet was disrupted on ice with a probe sonicator. The brain lysate was centrifuged at 12,000*g* for 10 min at 4 °C with protein concentration quantified using a BCA assay. In a 96-well plate, 10 µl brain lysate was added and adjusted to 40 µl with Glucosylceramidase assay buffer. Then 20 µl substrate was added and incubated at 37 °C for 30 min in dark. The reaction was stopped with 100 µl stop buffer and the fluorescence was read at 360 nm excitation 445 nm emission.

High pressure liquid chromatography. To estimate striatal monoamine levels the mice were deeply anesthetized by CO_2, quickly decapitated, and the striata dissected on ice, homogenized in 0.1 M perchloric acid, centrifuged, filtered, and stored at -80°C until analysis for monoamine content. Standard solutions of dopamine hydrochloride were prepared in 0.1 M perchloric acid to obtain final standard concentrations of 200, 100, 50, 10, 5, 2 and 1 ng/ml. Calibration curves were obtained with the Chromeleon software through linear regression of peak area versus concentration. The analysis was performed on HPLC-ECD system (Dionex Ultimate 3000, ThermoFisher Scientific, Waltham, MA, USA). The separation was performed on a C18 reversed-phase column at 30 °C. The mobile phase (75 mM monobasic sodium phosphate, 2.2 mM OSA, 100 µL/L TEA, 25 µM EDTA, and 10% acetonitrile (*v/v*), pH 3.0), was pumped at a flow rate of 0.4 mL/min. The first and second analytical cells were set to −100 mV and +300 mV, respectively. Processed samples were thawed on ice about an hour before analysis, placed in the autosampler, and kept at 5 °C before injection. Chromatograms were acquired with Dionex Chromeleon 7 software over an acquisition time of 55 min. Analyte concentrations in tissue samples were expressed as ng/mg of frozen tissue.

Cell culture - FA sample preparation, profiling and analysis (OmegaQuant). Cell culture total fatty acid composition was analyzed at OmegaQuant by gas chromatography (GC) with flame

ionization detection. Cells were harvested and resuspended in methanol. The methanol and pellet samples were transferred into their own respective screw-cap glass vial and dried down using an Organomation Associates Inc. nitrogen evaporator. After drying the methanol and pellet samples, 14 % boron trifluoride - methanol (Sigma-Aldrich, St. Louis, MO) and hexane (EMD Millipore Chemicals, USA) were added. The vials were capped, vortexed, and centrifuged to separate the layers. GC-FID was carried out using a GC2010 Gas Chromatograph (Shimadzu Corporation, Columbia, MD) equipped with a Supelco SP2560 fused silica capillary column (100m x 0.25 mm internal diameter x 0.2 um film thickness; Supelco, Bellefonte, PA). Fatty acids were identified by comparison with a standard mixture of fatty acids characteristic of RBC (GLC OQ-A, NuCheck Prep, Elysian, MN), which was also used to determine individual fatty acid calibration curves. Fatty acids were normalized to total fatty acid counts (% total fatty acid composition).

Lipid sample preparation, profiling and analysis (Lipotype). Mass spectrometry-based lipid analysis was performed by Lipotype GmbH (Dresden, Germany) as described(108). Lipids were extracted using a two-step chloroform/methanol procedure(109),(110). Samples were spiked with internal lipid standard mixture containing: cardiolipin 14:0/14:0/14:0/14:0 (CL), ceramide 18:1;2/17:0 (Cer), diacylglycerol 17:0/17:0 (DAG), hexosyl-ceramide 18:1;2/12:0 (HexCer), lyso-phosphatidate 17:0 (LPA), lyso- phosphatidylcholine 12:0 (LPC), lyso-phosphatidylethanolamine 17:1 (LPE), lyso-phosphatidylglycerol 17:1 (LPG), lyso-phosphatidylinositol 17:1 (LPI), lyso-phosphatidylserine 17:1 (LPS), phosphatidate 17:0/17:0 (PA), phosphatidylcholine 17:0/17:0 (PC), phosphatidylethanolamine 17:0/17:0 (PE), phosphatidylglycerol 17:0/17:0 (PG), phosphatidylinositol 16:0/16:0 (PI), phosphatidylserine 17:0/17:0 (PS), cholesterol ester 20:0 (CE), sphingomyelin 18:1;2/12:0;0 (SM) and triacylglycerol 17:0/17:0/17:0 (TAG). After extraction, the organic phase was transferred to an infusion plate and dried in a speed vacuum concentrator. First step dry extract was resuspended in 7.5 mM ammonium acetate in chloroform/methanol/ propanol (1:2:4,V:V:V) and second step dry extract in 33% ethanol solution of methylamine in chloroform/methanol (0.003:5:1; V:V:V). All liquid handling steps were performed using Hamilton Robotics STARlet robotic platform with the Anti Droplet Control feature for organic solvents pipetting. Samples were analyzed by direct infusion on a QExactive mass spectrometer (ThermoScientific) equipped with a TriVersa NanoMate ion source (Advion Biosciences). Samples were analyzed in both positive and negative ion modes with a resolution of Rm/z = 200 = 280,000 for MS and Rm/z = 200 = 17,500 for MSMS experiments, in a single acquisition. MSMS was triggered by an inclusion list encompassing corresponding MS mass ranges scanned in 1Da increments. Both MS and MSMS data were combined to monitor CE, DAG, and TAG ions as ammonium adducts; PC, PC O-, as acetate adducts; and CL, PA, PE, PE O-, PG, PI, and PS as

deprotonated anions. MS only was used to monitor LPA, LPE, LPE O-, LPI, and LPS as deprotonated anions; Cer, HexCer, SM, LPC, and LPC O- as acetate adducts. Data were analyzed with in-house developed lipid identification software based on LipidXplorer(111). Data post-processing and normalization were performed using an in-house developed data management system. Only lipid identifications with a signal-to-noise ratio >5, and a signal intensity 5-fold higher than in corresponding blank samples were considered for further data analysis.

FA sample preparation, profiling, and analysis (OmegaQuant). Cell culture total fatty acid composition was analyzed by gas chromatography (GC) with flame ionization detection. Cells were harvested and resuspended in methanol. The methanol and pellet samples were transferred into their own respective screw-cap glass vial and dried down using an Organomation Associates Inc. nitrogen evaporator. After drying the methanol and pellet samples, 14 % boron trifluoride - methanol (Sigma-Aldrich, St. Louis, MO) and hexane (EMD Millipore Chemicals, USA) were added. The vials were capped, vortexed, and centrifuged. GC-FID was carried out using a GC2010 Gas Chromatograph (Shimadzu Corporation, Columbia, MD) equipped with a Supelco SP2560 fused silica capillary column (100m x 0.25 mm internal diameter x 0.2 um film thickness; Supelco, Bellefonte, PA). Fatty acids were identified by comparison with a standard mixture of fatty acids characteristic of RBC (GLC OQ-A, NuCheck Prep, Elysian, MN), which was also used to determine individual fatty acid calibration curves. Fatty acids were normalized to total fatty acid counts (% total fatty acid composition).

Confocal Imaging and Image J analyses. Confocal microscopy was conducted with a Zeiss LSM710 confocal microscope (Karl Zeiss, Germany) Exposure time, gain, and light intensity were the same for all prepared slides. For confocal images of TH, LAMP1, pS129 and DAPI, each image was color-balanced. An ImageJ plug-in called "Colocalization highlighter" created a mask of LAMP1 overlapped with TH or pS129 αS pixels and binaries were used for the particle size analysis. For TFEB analysis, a Leica TCS SP8 STED 3X microscope (Leica Microsystems) and tile scans of SNpc region were made based on TH signal using the LAS X navigator software (Leica). Z-stacks of 15-20μm (z-step size 1.00μm) were made to include full-sized nuclei of TH+ neurons. All images were acquired using a HC PL APO CS2 40× 1.4 NA oil objective lens (zoom factor: 0.75), at a resolution of 2048 x 2048 pixels. For confocal images of TFEB, the same threshold was applied to all merged tile scans. Masks were generated for the DAPI channel, which

were overlayed on the TFEB images, which were made binary for the particle analysis. Analysis was repeated twice, analyzed and plotted by an unbiased experimenter.

Assessment of striatal DAergic fiber integrity. 25-µm-free floating sections were rinsed in TBS (0.15M NaCl, 0.1M Tris-HCl, pH 7.5) and endogenous peroxidase activity quenched with 0.6% H_2O_2 in TBS for 30 min at RT. Unspecific protein binding was blocked with 3% normal donkey serum in TBS containing 0.3% Triton X-100. Sections were incubated with rabbit TH (AB152, 1:500, Millipore) over night at 4°C. Following 3 rinses with TBS, sections were incubated with secondary anti-rabbit (1:1000, Dianova 711-065-152) in TBS containing 3% normal donkey serum, washed, and subsequently transferred into ABC solution (1:500 in PBS; Vectastain Elite Kit, Vector Laboratories) for 1 h and visualized with 3,3'-diaminobenzidin (DAB). Brain sections across a +4.8 to +3.5 interaural range were chosen, referring to the Paxinos and Franklin mouse brain atlas(106). Images were converted to gray scale, and the mean gray value intensity was measured in the caudate/putamen (CPu) and in the adjacent corpus callosum (cc) to correct for signal background. Threshold settings were set identically in the dorsal striatum for all sections. Mean gray values were converted to uncalibrated optical density (UOD) using ImageJ 1.46r software (NIH). The UOD of TH signal in the CPu was calculated by the formula $CPu_{final(UOD)} = CPu_{(UOD) - } cc_{(UOD)}$ The integrated density was analyzed and plotted by an unbiased experimenter.
