# Supplementary material for: Stearoyl-CoA desaturase inhibition normalizes brain lipid saturation, α-synuclein homeostasis, and motor function in mutant Gba1-Parkinson mice
Source: JCI Insight. 2025 Jun 3;10(13):e188413. doi: 10.1172/jci.insight.188413 (PMC12288896; doi:10.1172/jci.insight.188413)

UNEDITET FULL-LENGTH BLOTS FOR:

Figure 1I

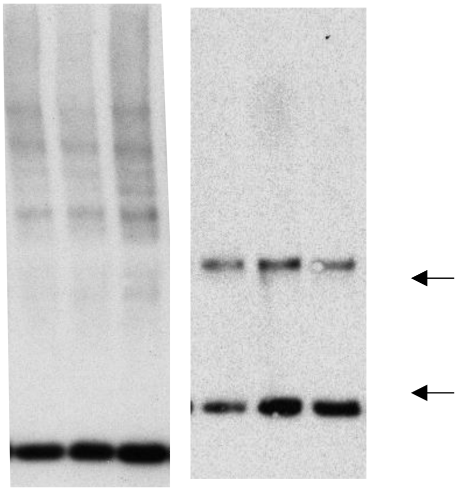

syn1 DJ-1

Figure 1L

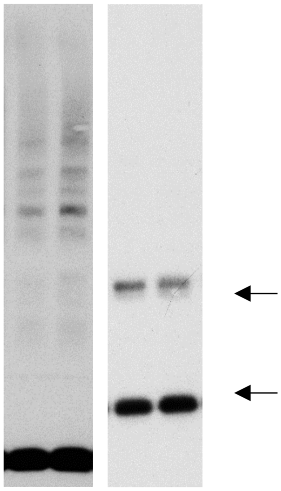

syn1 DJ-1

Figure 3B

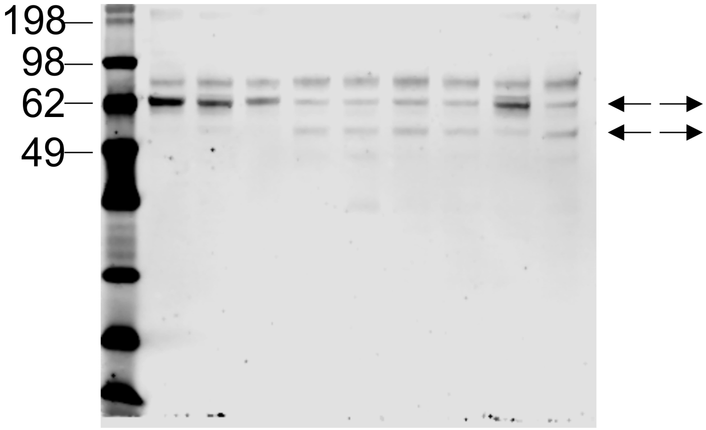

GCase

Figure 5A (cut; upper half)

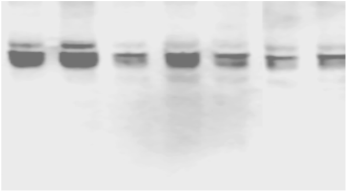

Figure 5A (cut; lower half)

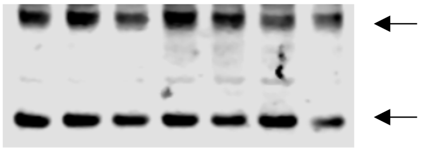

DJ-1

Figure 3B

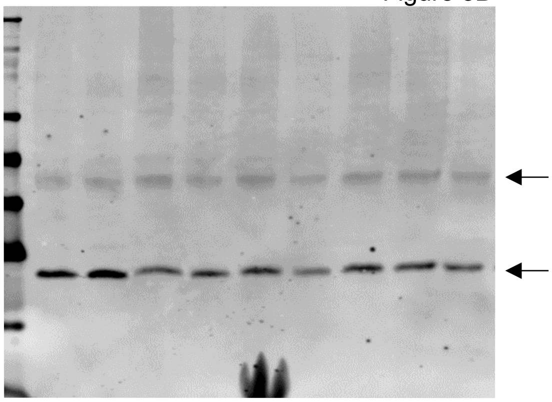

DJ-1

Figure 3B

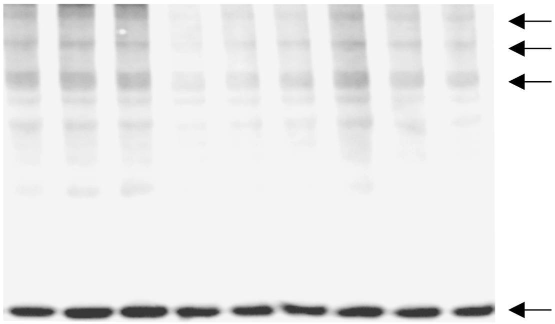

syn1

UNEDITET FULL-LENGTH BLOTS FOR:

Figure 3E, RIPA, cortex

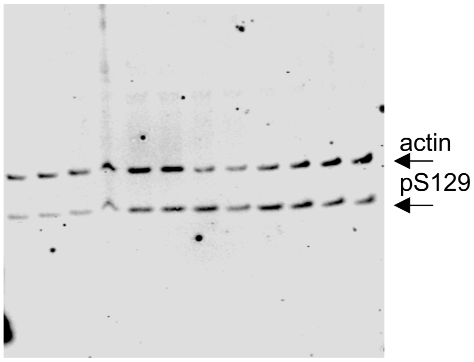

actin (RIPA), cortex
pS129 (RIPA), cortex

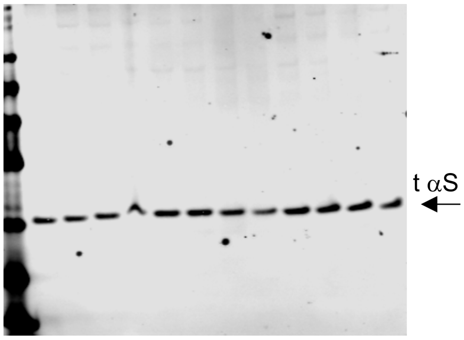

total αS (RIPA), cortex

Figure 3E, TBS, midbrain

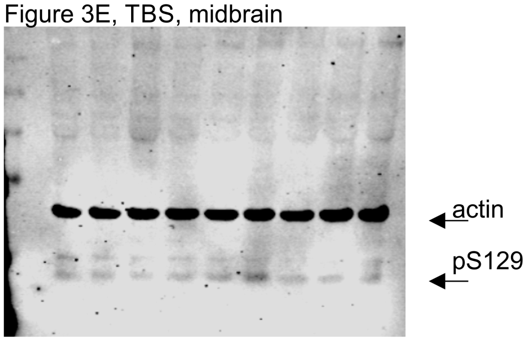

actin (TBS), midbrain
pS129 (TBS), midbrain

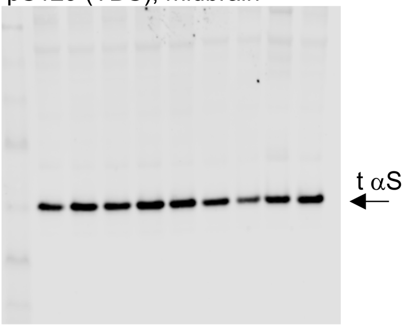

total αS TBS, midbrain

Figure 3E, RIPA, midbrain

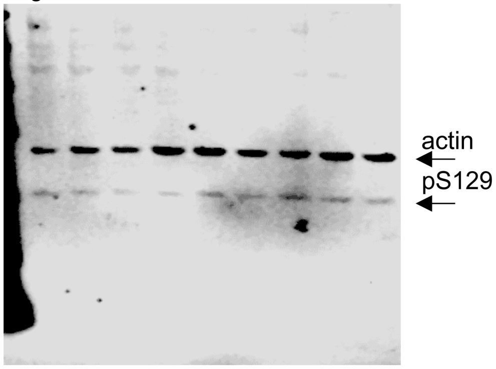

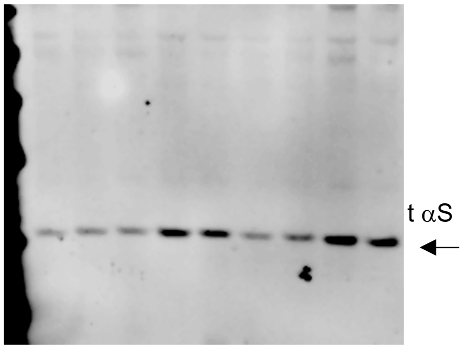

total αS (RIPA), midbrain

UNEDITED cut blots, containing only one specific signal (see page 2)

Figure 3E, TBS, cortex

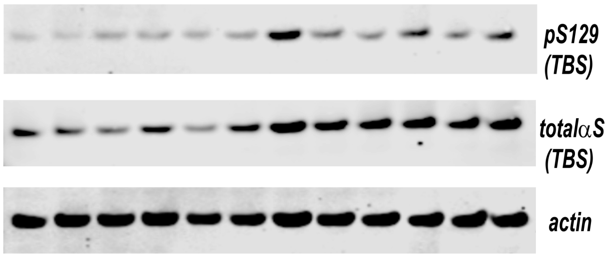

Figure 5B

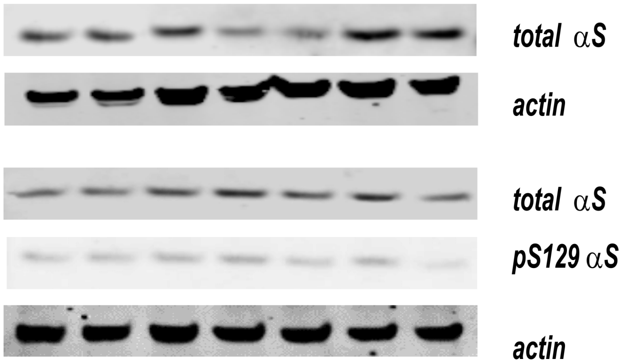

Figure 5C

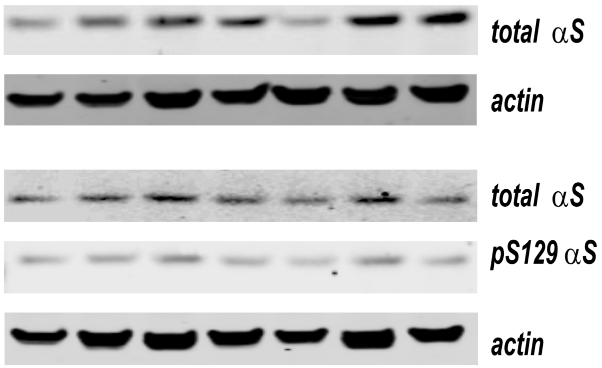

Supplement: Unedited blot and gel images [file jciinsight-10-188413-s192.pdf]
